# CD4 expression controls epidermal stem cell balance

**DOI:** 10.1038/s41598-025-87915-7

**Published:** 2025-02-04

**Authors:** Nadine Brandes, Heidi Hahn, Anja Uhmann

**Affiliations:** Institute of Human Genetics, Tumor Genetics Group, Universitätsmedizin Göttingen, Heinrich-Düker-Weg 12, 37073 Göttingen, Germany

**Keywords:** CD4, Epidermal stem cells, Interfollicular epidermis, Infundibulum, Wound healing, Ageing, Stem-cell niche, Skin stem cells, Ageing

## Abstract

The balance of stem cell populations is essential for the maintenance, renewal, and repair of the mammalian epidermis. Here, we report that CD4, which is a typical marker of helper T cells, monocytes, macrophages, and dendritic cells, is also expressed on murine K5^+^ keratinocytes. Lineage tracing of CD4^+^ cells reveals that their epidermal progeny has self-renewal abilities and clonogenic potential. The progeny of CD4^+^ epidermal cells contributes to epidermal renewal and progressively colonizes the interfollicular epidermis and hair follicles with age, thereby developing to all epidermal lineages. Wound healing studies furthermore show that the progeny of CD4^+^ epidermal cells accumulates at wound sites. Finally, using *CD4* knockout mice we demonstrate that CD4 expression is essential for maintaining fast-cycling epidermal stem cells during homeostasis and that CD4 loss mitigates the age-related decline in wound repair capacity. Collectively, our data support the conclusion that CD4 expression is required for long-term maintenance of the epidermal stem cell balance.

## Introduction

Adult stem cells (SCs) are characterized by their self-renewal capacity, high clonogenic potential, slow cycling activity, and the potential to generate differentiated progeny^1^. They are essential for homeostasis and regeneration of adult tissues. In the epidermis of the adult skin, SCs are located in the interfollicular epidermis (IFE) and in its appendages, such as the hair follicles and associated sebaceous glands. During homeostasis, each of these epidermal SC population behaves independently and maintains its own epidermal structure by dividing and differentiating^2^. Even the SC compartment of the basal layer of the IFE (BL-IFE) is composed of SCs that differ in their cycling behavior, survival, and response to injury^3–7^. Unlike the SCs of the BL-IFE, hair follicle SCs are not involved in IFE homeostasis, but they contribute to IFE recovery in response to wound healing processes^3^. The ability of the epidermis to recover from damage or stress decreases with age. While young skin still contains a heterogeneous population of epidermal fast- (fc) and slow-cycling (sc) SCs^5,7,8^, fc-SCs are depleted in aged skin and the available space is colonized by sc-SCs^8,9^. Simultaneously, the lineage differentiation of sc-SCs is impaired, and the wound healing capacity is reduced^8,9^. However, the complexity of epidermal turnover, the ability to recover from stress and injury, and the age-related changes in skin morphology and composition are still not fully understood.

We have previously reported that CD49f^+^ CD34^+^SCA-1^+^ epidermal cells, which represent a subpopulation of hair follicle SC in the murine back skin^10^, co-express CD4^11^. Using a CD4Cre-based in vivo depletion strategy we have furthermore demonstrated that CD4Cre-targeted cells co-express K5^+^ and can give rise to basalioma^12^. This suggests that CD4 is expressed in an epidermal SC population, at least in murine skin. However, neither the epidermal niche nor the role or the essentiality of this population for epidermal integrity has been investigated.

We report here that CD4 expression is required for the maintenance of fc-SCs. Lineage tracing of CD4^+^ cells furthermore shows that their epidermal progeny (i) has self-renewal capacity and high clonogenic potential, (ii) progressively colonizes the IFE and the hair follicle with mouse age, (iii) grows as all hair follicle lineages, (iv) accumulates at wound near epidermal areas and (v) that CD4 loss improves age-related worsening of wound repair.

## Results

### CD4 expression controls proliferation in infundibulum and IFE of the adult back skin

We recently demonstrated that active Hedgehog signaling leads to basalioma formation from CD4-expressing cells^11,12^. This indicates a function of CD4 in basalioma progenitor cells, most likely in epidermal SC. Based on this assumption, *CD4* loss in epidermal cells should result in a considerable skin phenotype. Due to the lack of a conditional *CD4KO* mouse model, we here tested this hypothesis by analyzing conventional *CD4KO* mice (subsequently referred to as *CD4KO*). This mouse strain is characterized by a block in thymic CD4^+^ T-cell development and a Class II restricted deficit in helper T-cell activity due to a targeted *Cd4* mutation^13,14^. Previous analyses of the skin of young and middle-aged *CD4KO* mice (8–30 weeks) did not report on a conspicuous skin phenotype^15–19^. However, aged *CD4KO* mice have not yet been investigated, and thus any potential skin phenotype in these mice remains unknown. Here, we conducted detailed skin morphology analyses of the epidermal back skin (EBS) and epidermal tail skin (ETS) of 8 (young), 31 (middle-aged) and 80 (aged) weeks-old *CD4KO* and age-matched wild-type (wt) control mice (cntl) (for analyzed animal numbers see Suppl. Tables S1 and S2).

**Fig. 1:**
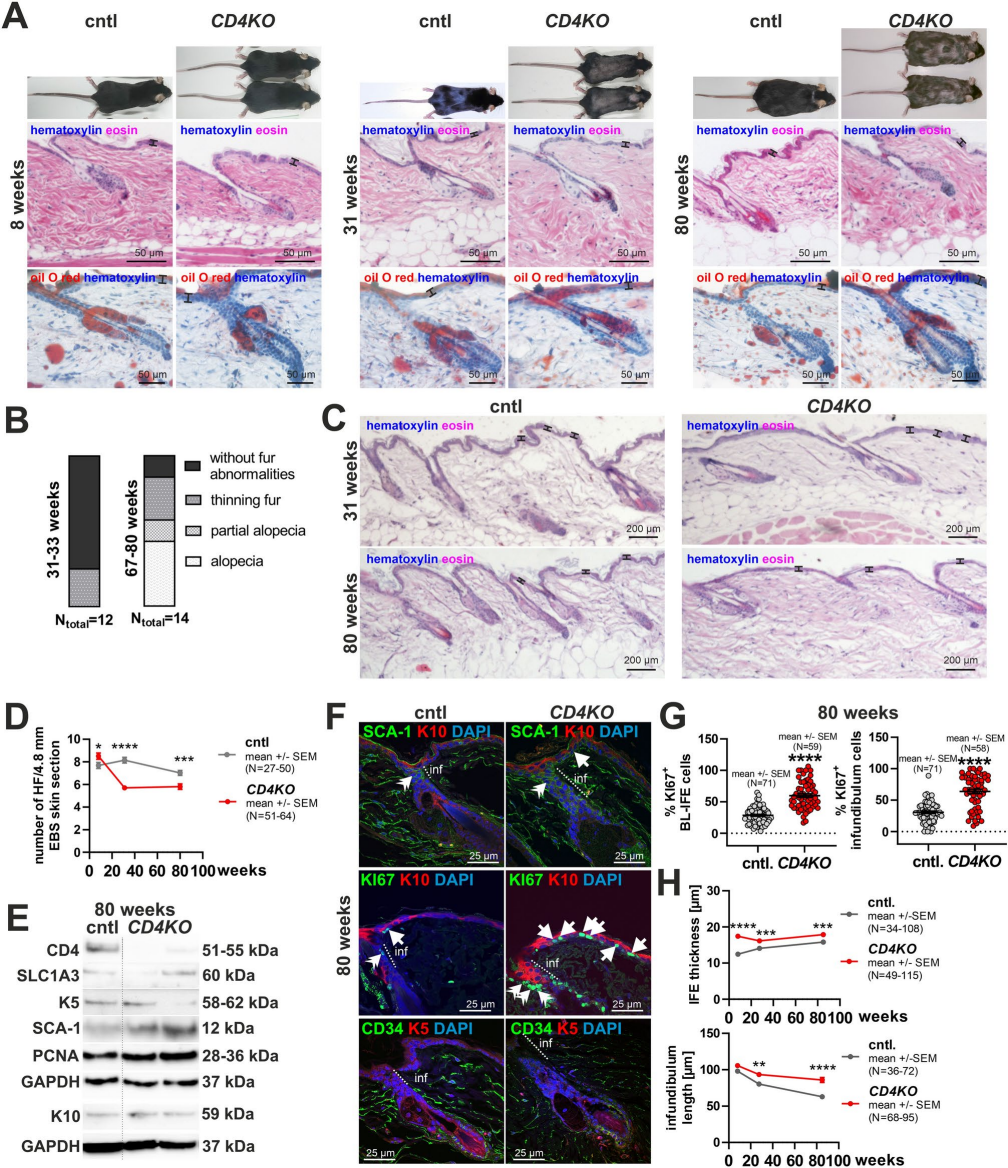
The EBS phenotype of *CD4KO* mice. Analyses of the EBS of 8, 31 and/or 80 weeks-old *CD4KO* and corresponding cntl mice. Aged *CD4KO* mice show alopecia of the back skin fur with reduced numbers of hair follicles (**A**–**D**), decreased expression levels of the IFE markers K5 and SLC1A3 (**E**), increased expression levels of the BL-IFE and INF marker SCA-1 (**E**,**F**) and the proliferation markers PCNA (**E**) and KI67 (**F**,**G**), thicker IFE and longer INF (**H**). (**A**) Macroscopic and microscopic hematoxylin/eosin and oil O red/hematoxylin analyses; (**B**) quantification of *CD4KO* back skin fur abnormalities; (**C**,**D**) hematoxylin/eosin analyses and quantification of the number of hair follicles per visual field (4.8 mm skin) of hematoxylin/eosin-stained paraffin sections; (**E**) Western blot analyses; (**F**) immunostainings of paraffin sections against SCA-1/K10, KI67/K10 and CD34/K5; (**G**) quantification of KI67^+^ BL-IFE and INF cells; (**H**) IFE thickness and INF length of cntl and *CD4KO* EBS of 8, 31 and/or 80 weeks-old mice. Protein bands in (**E**) were cropped from the original blots shown in Supplemental Fig. S12. Black bars in (**A**,**C**) visualize the IFE thickness. Nuclei in (F) were visualized with DAPI. White double arrowheads: SCA-1^+^ or KI67^+^ INF cells; white arrows: SCA-1^+^ or KI67^+^ IFE cells. inf: infundibulum. N in (**D**,**G**,**H**) corresponds to the number of measured points. *P < 0.05, **P < 0.01, ***P < 0.001, **** P < 0.0001. See Suppl. Tables S1, S3 and S6 for animal numbers and means, SEM and P values.

While all young *CD4KO* mice macroscopically looked normal, 25% of the middle-aged, and 85.7% of the aged *CD4KO* mice developed partial to distinct alopecia of the back skin fur (Fig. 1A–C; Suppl. Tables S1, S3). This suggests a role of CD4 in epidermal SC function upon skin aging. Interestingly, all middle-aged and aged *CD4KO* mice had reduced numbers of hair follicles (Fig. 1C,D). However, this was not accompanied by an abnormal immune cell composition (Supp. Fig. S1) or obvious histological abnormalities in telogen hair follicles, IFE or sebaceous glands (Fig. 1A,C). The EBS from aged *CD4KO* mice had increased expression levels of the BL-IFE and INF marker stem cell antigen-1 (SCA-1) and the proliferation marker proliferating cell nuclear antigen (PCNA), whereas the BL-IFE markers K5 solute carrier family 1 member 3 (SLC1A3) and the suprabasal marker K10 were slightly decreased (Fig. 1E). Immunostainings showed elongated SCA-1^+^ INF and increased numbers of KI67^+^ BL-IFE and KI67^+^ INF cells in aged *CD4KO* mice (Fig. 1F,G). This was accompanied by a significantly thicker IFE at all ages and longer INF in aged *CD4KO* mice (Fig. 1H). Thus, these observations were most likely causative for the enhanced SCA-1 and PCNA expression levels detected by Western blot (Fig. 1E). Although the expression patterns of K5 and K10 appeared similar between *CD4KO* and cntl EBS (Fig. 1F), the K5 expression level seems to be decreased in *CD4KO* as judged by Western blot (Fig. 1E). Finally, the expression pattern of the hair follicle SC (bulge) marker CD34 was similar in telogen hair follicles in *CD4KO* and cntl EBS (Fig. 1F).

*CD4KO* mice show a deficit in helper T-cell activity^13,14^. To exclude that defective immune cells cause the observed epidermal phenotype of *CD4KO* mice we next analyzed in vitro cultured keratinocytes isolated from the EBS of *CD4KO* and cntl mice. At in vitro 3D conditions, number and size of organoids formed from EBS cells of young *CD4KO* mice did not differ from the corresponding cntl (Suppl. Fig. S2A–E). Cells from middle-aged cntl mice formed significantly less (Suppl. Fig. S2A,B) but larger organoids (Suppl. Fig. S2C) than from corresponding cntl. This suggests that middle-aged wt EBS contains fewer SCs with a higher SC capacity than that of young wt mice. In contrast, keratinocytes from middle-aged *CD4KO* mice developed similar numbers, but significantly smaller organoids (Suppl. Fig. S2A,B) compared to young *CD4KO* and middle-aged cntl mice (Suppl. Fig. S2C). This shows that CD4 loss counteracts age-related SCs loss but reduces the SC capacity of EBS SCs. Neither the expression of K5, SCA-1 and PCNA (Suppl. Fig. S2D,E) nor the capacity of *CD4KO* EBS cells to form hair follicles after hypodermal transplantation into *Foxn1*^*nu/nu*^ mice was altered (Suppl. Fig. S2F).

Together with the fact that aged *CD4KO* mice show an increased proliferation of BL-IFE and INF, these data show that CD4 is involved in age-related SC loss and quiescence in the EBS.

### CD4 expression controls scale/interscale balance of the IFE in the adult tail skin

**Fig. 2:**
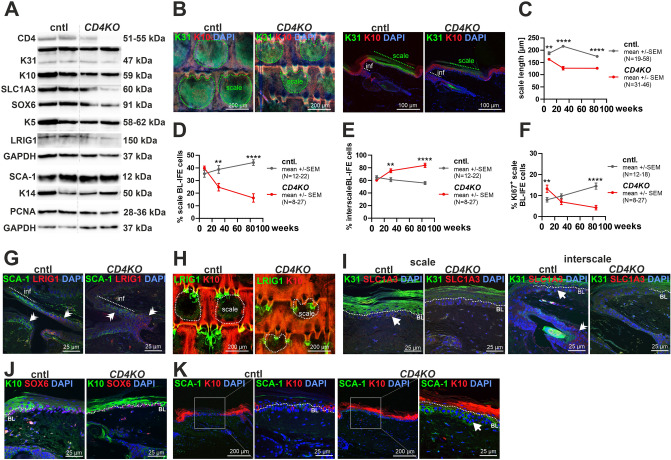
The ETS phenotype of *CD4KO* mice. Analyses of the EBS of 8, 31 and/or 80 weeks-old *CD4KO* and corresponding cntl mice. Aged *CD4KO* mice show decreased SLC1A3 and PCNA expression levels (**A**), irregular scale/interscale structure (**B**), with significantly shorter scales (**C**), decreased number of BL-IFE cells below the K31^+^ scales (**D**), increased number of BL-IFE cells below K10^+^ interscales (**E**) and lower proliferation level scales BL-IFE cells (**F**). (**A**) Western blot analyses of ETS from 80 weeks-old cntl and *CD4KO* mice. (**B**,**G**–**M**) immunostained ETS sheets (B left, H) or paraffin sections (B right, G,I–M) stained with antibodies against K31/K10 (**B**), SCA-1/LRIG1 (G), LRIG/K10 (H), K31/SLC1A3 (I), K10/SOX6 (J) and SCA-1/K10 (K) from 80 weeks-old cntl and *CD4KO* mice. (**C**–**F**) Quantification of the scale length (**C**), percentage of all BL-IFE cells of scales (**D**) and interscales (**E**) and KI67^+^ BL-IFE cells of scales (**F**) of 8, 31 and 80 weeks-old cntl and *CD4KO* mice. Protein bands in (**A**) were cropped from the original blots shown in Supplemental Fig. S13. Nuclei were visualized with DAPI. Dotted lines in (B,H) define the scale borders and in (I-K) the basal layer (BL). White double arrowhead: SCA-1^+^, LRIG1^+^ or SLC1A3^+^ hair follicle cells; white arrows: SLC1A3^+^, SCA-1^+^ or LEF1^+^ BL-IFE cells. White boxes: zoom-in areas. inf: infundibulum. N in (**C**–**F**) corresponds to the number of measured points. **P < 0.01, ****P < 0.0001. See Suppl. Tables S2 and S7 for animal numbers and means, SEM and P values.

*CD4KO* ETS macroscopically looked normal, even in aged animals (Suppl. Fig. S3A). No abnormal immune cell invasion (Suppl Fig. S1) or histological abnormalities were found in hair follicles, IFE or sebaceous glands (Suppl. Fig. S3A), and K31, K10, SOX6, K5, LRIG1, SCA-1 or K14 protein levels were not altered (Fig. 2A, Suppl. Fig. S3B) in aged *CD4KO* ETS. However, SLC1A3 and PCNA expression levels were significantly decreased in aged *CD4KO* compared to cntl mice (Fig. 2A, Suppl. Fig. S3B). This suggests an altered epidermal homeostasis in *CD4KO* ETS. In fact, middle-aged, and aged *CD4KO* mice had an irregular scale/interscale structure (Fig. 2B, Suppl. Fig. S4A,B) with significantly shorter (Fig. 2C) and thicker (Suppl. Fig. 4C) K31^+^ scales compared to age-matched cntl mice. Concomitantly, the numbers of BL-IFE cells below the K31^+^ scales were decreased (Fig. 2D), whereas those of the K10^+^ interscales were increased in *CD4KO* compared to age-matched cntl mice (Fig. 2E). The proliferation rate of BL-IFE cells in the scales was higher in young *CD4KO* mice but decreased to an equal or lower level in middle-aged and aged *CD4KO* mice, respectively (Fig. 2F, Suppl. Fig. S4D). In contrast, the proliferation of BL-IFE cells of the interscales did not differ between *CD4KO* and cntl mice (Suppl. Fig. S4E,F), although the interscales of young and aged *CD4KO* mice were thicker (Suppl. Fig. S4G). Like in the EBS, all INF were longer at any age and showed a higher proliferation rate in middle-aged and old *CD4KO* ETS (Suppl. Fig. S4I). However, the overall expression level or protein distribution of SCA-1 or the INF/JZ marker LRIG1 at the hair follicles was not altered in aged *CD4KO* ETS (Fig. 2G,H).

Wt ETS consists of fc-SCs below the scale and sc-SCs below the interscale compartment^5,7,20^. Normally, scale and interscale BL- and suprabasal IFE are strictly separated and temporary loss of one of both SC populations results in temporary mixture of the compartments due to an inefficient long-term maintenance in their atypical territory^5^. In *CD4KO* mice, this balance apparently is disturbed. Thus, scale BL-IFE cells of young *CD4KO* mice proliferate significantly more compared to cntl mice (Fig. 2F). With increasing age, their proliferative capacity decreases and falls below that of the cntl (Fig. 2F). Together with the reduced scale size in middle-aged and aged *CD4KO* mice (Fig. 2C), this suggests that in aged *CD4KO* mice fc-SC are depleted and that the scale compartment is overgrown by the interscales and the respective sc-SCs. Consistently, the expression area of the scale markers K31 (Fig. 2A,I) and SLC1A3 (Fig. 2A,I; Suppl. Fig. S3B) shrunk and that of the interscale BL-IFE marker SOX6 expanded (Fig. 2J) in aged *CD4KO* mice. Additionally, aged *CD4KO* ETS showed a broadening of SCA-1 expression in the BL-IFE (Fig. 2K).

Mice with WNT signaling inactivation in K14^+^ keratinocytes due to the depletion of lymphoid enhancer-binding factor-1 (LEF1) also have smaller scales^21^. However, we did not observe any changes in LEF1 (Suppl. Fig. S4J) or b-CATENIN (Suppl. Fig. S4K) distribution pattern in aged *CD4KO* ETS. LRIG1 depletion is also associated with altered scale/interscale morphology^22^, but LRIG1 expression in aged *CD4KO* ETS was also unaltered (Fig. 2A,G,H).

These data show that in aged *CD4KO* ETS, the SCL1A3^+^/K31^+^ scale compartments are overgrown with SOX6^+^ sc-SCs and their corresponding K10^+^ suprabasal cells. Since INF cells also contribute to IFE homeostasis^23^, their increased proliferation rate (see above) may also influence the overgrowth of the interscale in aged *CD4KO* ETS.

### CD4^+^ cells of the INF and IFE are long-term survivors colonizing hair follicle and IFE

The epidermal phenotype of *CD4KO* mice suggests that CD4 expression is crucial for maintaining epidermal homeostasis, particularly in aged mice. However, we did not observe an accumulation of immune cells in *CD4KO* skin (see Suppl. Fig. 1), which could be responsible for the altered SC homeostasis. Thus, we hypothesized that our recently identified CD49f^+^CD34^+^SCA-1^+^ cells, which most likely represent a subpopulation of hair follicle cells^10^ and which stain positive for anti-CD4 antibodies^11^, play a role in the epidermal phenotype of *CD4KO* mice.

**Fig. 3:**
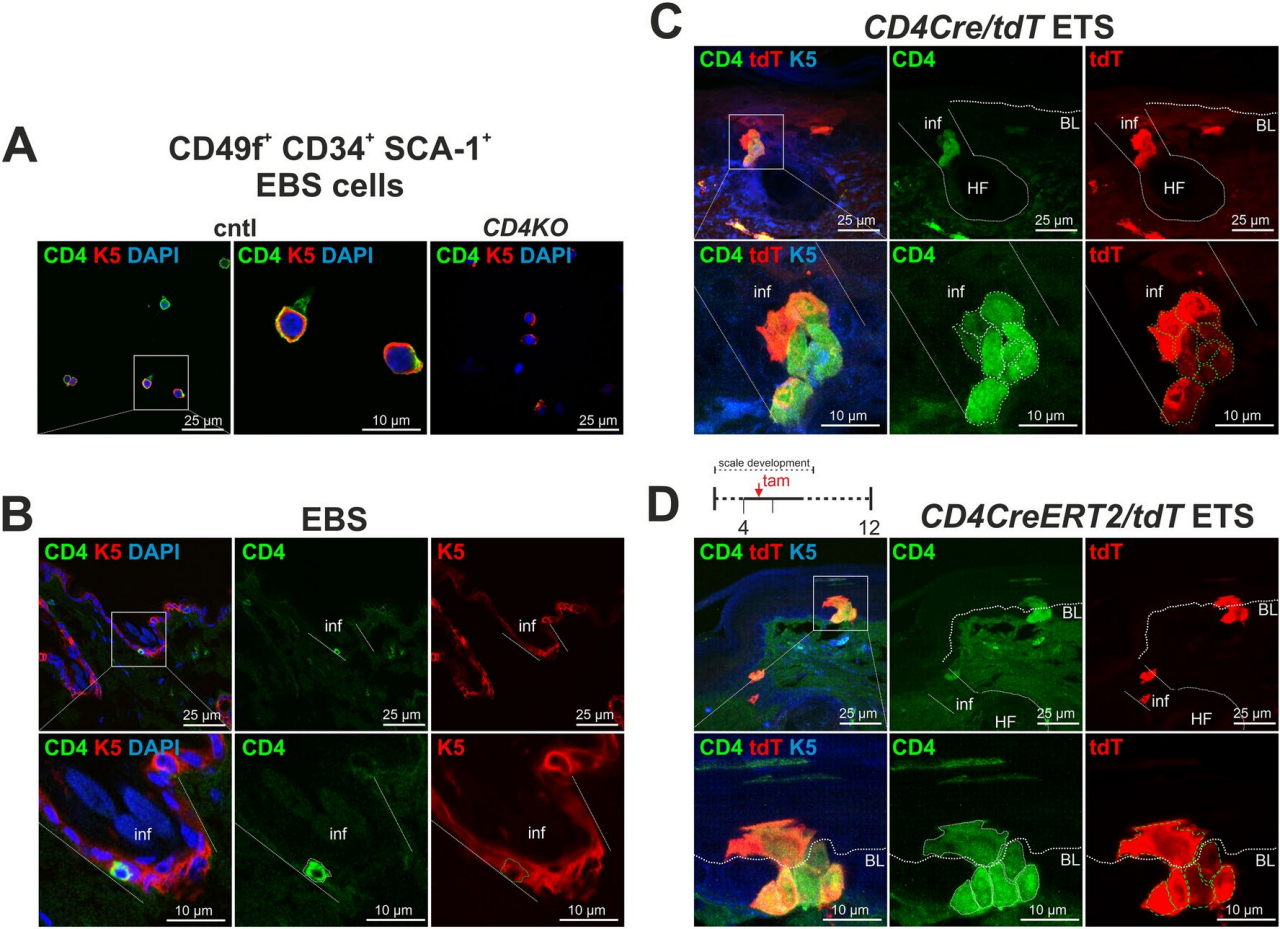
K5^+^ epidermal cells can express CD4. Analyses of the CD4 expression in keratinocytes of the EBS and ETS of wt and *CD4Cre/tdT* and *CD4CreERT2/tdT* lineage tracing mice. CD4 is expressed in K5^+^ keratinocytes of wt EBS (**A**,**B**) and *CD4Cre/tdT* (**C**) and *CD4CreERT2/tdT* ETS (**D**). (**A**) Anti-CD4/anti-K5 antibody stained cytospun CD49f^+^CD34^+^SCA-1^+^ EBS cell of 5 weeks-old cntl and *CD4KO* mice. (**B**–**D**) Anti-CD4/K5 immunofluorescent analyses of wt EBS paraffin sections (**B**) and *CD4Cre/tdT* (**C**) or *CD4CreERT2/tdT* (**D**) ETS cryosections. tdT^+^ cells were detected in (**C**,**D**) by native tdT expression. Tamoxifen (tam)-regime of *CD4CreERT2/tdT* mice is depicted in (**D**). Nuclei were visualized with DAPI. White arrows: CD4^+^ K5^+^ cells of the infundibulum (inf) or the basal layer (BL). White boxes: zoom-in area. HF: hair follicle. Skin samples were collected from 11 to 12 weeks-old mice.

First, cytospun FACsorted CD49f^+^CD34^+^SCA-1^+^ cells from wt EBS were verified to express CD4 and the keratinocyte SC marker K5 (Fig. 3A). Immunofluorescent stainings of wt EBS sections attested the existence of K5^+^ CD4^+^ INF cells (Fig. 3B). Quantification of CD49f^+^ CD4^+^ keratinocytes furthermore demonstrated the previously reported relative abundance of these cells with 0.5–1.4% of all CD49f^+^ EBS keratinocytes in young and middle-aged wt mice^11^ (Suppl. Fig. S5A,B, Suppl. Table S8). 83–85% of the cells co-expressed CD34 and SCA-1, and only 2–10% or 0.1–0.2% were CD34^+^ bulge or SCA-1^+^ BL-IFE cells, respectively (Suppl. Fig. S5A,C,D, Suppl. Table S8).

**Fig. 4:**
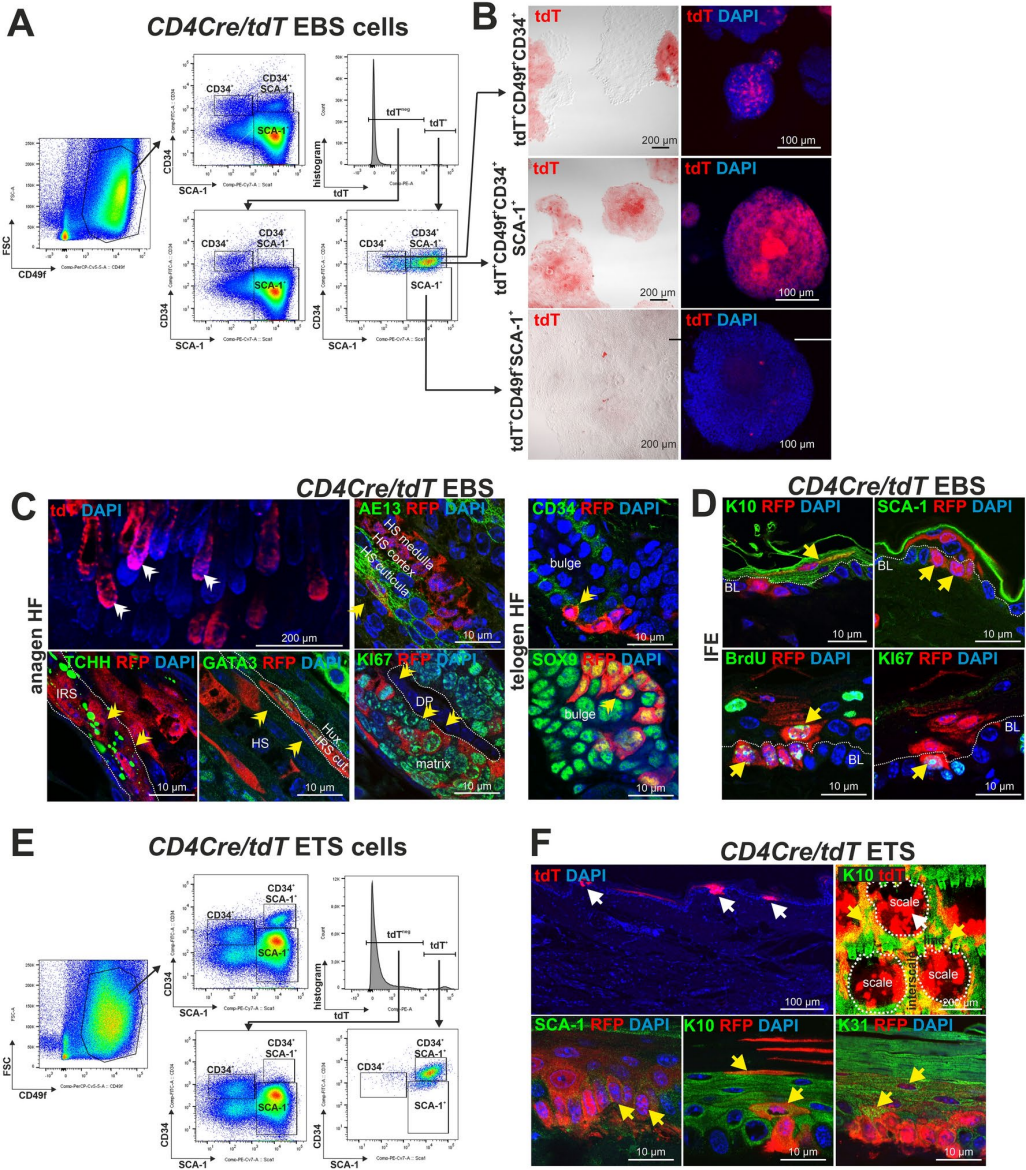
The progeny of CD4^+^ cells preferentially grows in hair follicles of EBS and in the IFE of ETS. Analyses of the progeny of CD4^+^ keratinocytes in the EBS and ETS of *CD4Cre/tdT* lineage tracing mice. The majority of progeny in the EBS and ETS co-express CD49f, CD34 and SCA-1 (**A**,**E**). In the EBS, they develop to bulge (tdT^+^ CD49f^+^CD34^+^) and rarely to BL-IFE cells (tdT^+^ CD49f^+^SCA-1^+^) (**A**). In vitro cultured tdT^+^CD49f^+^CD34^+^SCA-1^+^ and tdT^+^ CD49f^+^CD34^+^ bulge cells retained the tdT labeling and grow as large clones, whereas tdT^+^ CD49f^+^SCA-1^+^ BL-IFE cells lost it (**B**). In telogen hair follicles, the progeny develops to CD34^+^ or SOX9^+^ bulge cells. In anagen hair follicle, they grow in all layers of the root sheath (**C**). In the IFE they develop to K10^+^ IFE, SCA-1^+^ or proliferating BL-IFE cells (**D**). In the ETS, the progeny of CD4^+^ keratinocytes develop only rarely to bulge and mainly to BL-IFE cells (**E**). Here they colonize the scale, interscale, and line subregions and grow as SCA-1^+^ BL-IFE and K10^+^ or K31^+^ keratinocytes (**F**). (**A**,**E**) CD49f/FSC-plotted flow cytometric data of EBS (A) and ETS cells (E) of 70 to 80 weeks-old *CD4Cre/tdT* mice. All CD49f^+^ or tdT^neg^ CD49f^+^ or tdT^+^ CD49f^+^ keratinocytes were SCA-1/CD34-plotted for determination of CD34^+^ bulge, SCA-1^+^ IFE and CD34^+^SCA-1^+^ epidermal cells (see^11,12^). tdT^+^ cells were detected in the PE-channel. (**B**) Native tdT expression analyses of adherently, feeder-free growing (passage 1) and 3D-cultured (passage 2) tdT^+^CD49f^+^CD34^+^, tdT^+^CD49f^+^CD34^+^SCA-1^+^ and tdT^+^CD49f^+^SCA-1^+^ EBS cells from a 71 weeks-old *CD4Cre/tdT* mouse. (**C**,**D**,**F**) Fluorescent analyses of EBS (C;D) and ETS (F) of 25 to 60 weeks-old *CD4Cre/tdT* mice. Anagen and telogen tdT^+^ hair follicle (C) and tdT^+^ IFE (F) cells were detected by native tdT expression in an EBS whole mount (C), in unstained (F, top left), in an anti-K10 stained ETS sheet (F, top right) or in paraffin sections stained with antibodies against RFP and the hair cortex marker AE13, the inner root sheath markers trichohyalin (TCHH) and GATA3, KI67, CD34, SOX9, K10, SCA-1, BrdU, KI67 or K31. Nuclei were visualized with DAPI. White double arrowheads: tdT^+^ hair follicle cells; yellow double arrowheads: double positive tdT^+^ hair follicle cells; white arrows: tdT^+^ IFE cells, yellow arrows: double positive tdT^+^ IFE cells. White dotted lines in (D,H,I,J) define the basal layer (BL) and in (G) the tail skin scales. DP: dermal papillae; HS: hair shaft; IRS: inner root sheath; Hux: Huxley’s layer; IRS cut.: IRS cuticula.

Since these data indicate that K5^+^ EBS keratinocytes can express CD4 we next traced the progeny of CD4^+^ cells (tdT^+^) in *CD4Cre/tdT* and tamoxifen (tam)-inducible *CD4CreERT2/tdT* mice. These approaches showed that CD4^+^ K5^+^ epidermal cells co-express the lineage marker tdT^+^ and also grow in the INF and the basal layer of the ETS (Fig. 3C,D). Flow cytometric analyses furthermore revealed that, like CD4^+^ EBS cells^11^, the majority of tdT^+^ EBS and tdT^+^ ETS cells co-express CD49f, CD34 and SCA-1 (Fig. 4A,E). Remarkably, whereas in the EBS some tdT^+^ keratinocytes were CD49f^+^CD34^+^ bulge cells (Fig. 4A), in the ETS only a very small proportion of tdT^+^ ETS cells were CD49f^+^CD34^+^ bulge cell (Fig. 4E). Vice versa, in the EBS very few tdT^+^ cells were CD49f^+^SCA-1^+^ BL-IFE cells (Fig. 4A), whereas in the ETS a larger proportion of tdT^+^ cells were CD49f^+^CD34^+^ BL-IFE cells (Fig. 4E).

Differential gene expression analysis of FACsorted EBS cells further demonstrated that tdT^+^CD49f^+^CD34^+^ and tdT^+^CD49f^+^CD34^+^SCA 1^+^ cells were indistinguishable from their tdT^neg^ counterparts. However, while CD49f^+^CD34^+^ cells resembled a bulge cell identity, CD49f^+^CD34^+^SCA 1^+^ cells were more likely to be BL-IFE and suprabasal IFE cells (Suppl. Fig. S6). Upon in vitro culture of FACsorted EBS cells, tdT^+^ CD49f^+^CD34^+^ bulge and tdT^+ ^CD49f^+^CD34^+^SCA-1^+^ cells retained the tdT labelling and grew as large clones indicating their SC identity (Fig. 4B). In contrast, tdT^+ ^CD49f^+^SCA-1^+^ cells were lost during culture suggesting their more differentiated identity (Fig. 4B).

These observations were further substantiated by immunofluorescent analyses. In telogen *CD4Cre/tdT* EBS most tdT^+^ cells were found in the hair follicle and co-express the bulge markers CD34 or SOX9 (Fig. 4C). In anagen, these cells were found in all known hair types (Suppl. Fig. S7A,D) and all layers of the hair follicle root sheath (Fig. 4C). tdT^+^ cells grow as basal outer root sheath clones repopulating the hair follicle from the distal part, but also as complex clones colonizing both the inner and the outer root sheath and infiltrating the lower proximal cup and the dermal sheath (Fig. 4C; Suppl. Fig. S7B,C,E,F). A small number of tdT^+^ cells also colonize the IFE of EBS. Here they grow as suprabasal K10^+^ IFE cells, as SCA-1^+^ cells, or as actively proliferating BL-IFE cells as shown by BrdU incorporation after short term in vivo pulsing and anti-KI67 antibody staining (Fig. 4D).

In the ETS, tdT^+^ progeny colonizes the scale, the interscale, and the line subregions with the hair follicles of the IFE compartment (Fig. 4F). The identity of epidermal tdT^+^ cells as keratinocytes was evidenced by co-expression of the BL-IFE marker SCA-1 or the keratinocyte differentiation markers K10 or K31 (Fig. 4F). In addition, epidermal tdT^+^ cells were negative for the general immune cell marker CD45, the Langerhans’ cell marker Langerin and the melanocyte marker MelanA (Suppl. Fig. S8). This was similar in tam-induced *CD4CreERT2/tdT* mice (Suppl. Fig. S9).

Together these data show that a subpopulation of K5^+^ CD49f^+^CD34^+^SCA-1^+^ cells express CD4. The abundance of these CD4^+^ keratinocytes remain unaltered in young and middle-aged mice. The progeny of CD4^+^ keratinocytes accumulates with increasing age^12^, are long-term survivors and preferentially colonize the hair follicle in the EBS and the IFE of the line, and scale and interscale ETS.

### CD4^+^ epidermal cells contribute to epidermal wound healing

**Fig. 5:**
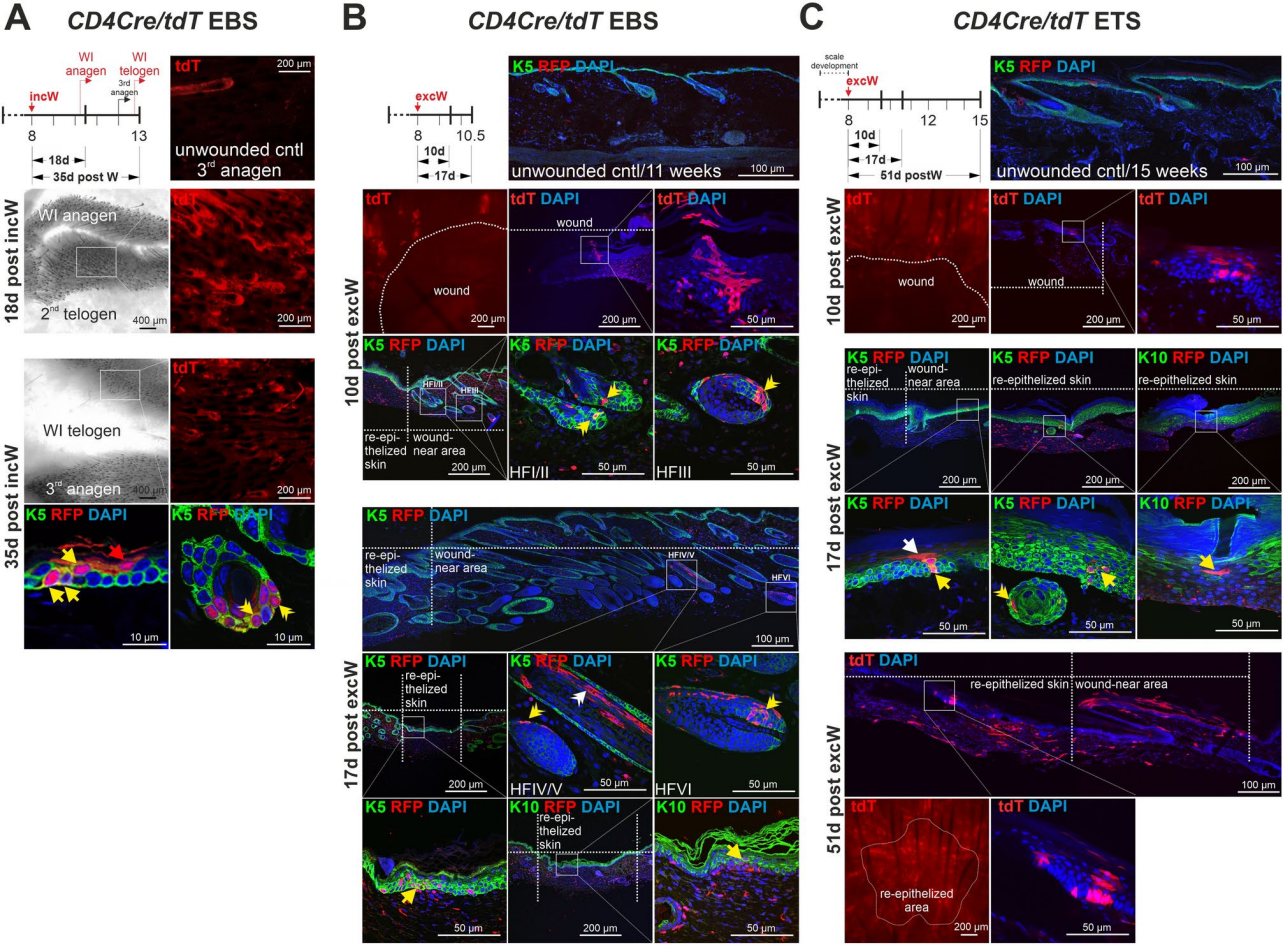
Wound-induced colonization of the hair follicle and/or the IFE with the progeny of CD4^+^ epidermal cells. Analyses of incisional (incW) and excisional wounding (excW) of *CD4Cre/tdT* lineage tracing mice. In *CD4Cre/tdT* mice, both wounding protocols result in tdT-labeling of wound-near hair follicle and IFE cells in the EBS (**A**,**B**) and of wound-near IFE cells of the ETS (**C**). (**A**–**C**) Experimental setup and (immune-) fluorescent analyses of unwounded (A-D), incW (A) and excW (B-D) of EBS (A,B) or ETS (C,D) of *CD4Cre/tdT* (A-C). (**A**) tdT^+^ hair follicle of the 3rd endogene anagen phase in the EBS of an unwounded *CD4Cre/tdT* mouse^12^ and in wound-near regions in *CD4Cre/tdT* EBS whole mounts and paraffin sections stained with anti-K5/anti-RFP antibodies 18d and 35d post incW. (**B**) tdT^+^ IFE and hair follicle cells in wound-near regions in an EBS whole mount, in EBS cryosections or anti-K5 or anti-K10 antibody-stained EBS paraffin sections 10d and 17d post excW or of unwounded skin. (**C**) tdT^+^ IFE and hair follicle cells in wound-near regions in ETS whole mounts and in ETS cryosections 10d and 51d post excW or anti-K5 or anti-K10 antibody-stained ETS paraffin sections 17d post excW or of unwounded skin. Nuclei were visualized with DAPI. tdT expression in paraffin sections was detected using an anti-RFP antibody. White double arrowheads: tdT^+^ hair follicle cells; yellow double arrowheads: double positive tdT^+^ hair follicle cells; white arrows: tdT^+^ IFE cells; yellow arrows: double positive tdT^+^ IFE cells; WI: wound-induced; postW: post wounding. White boxes: zoom-in areas.

CD4 is expressed in epidermal SCs, which are involved in epidermal wound healing^3^. Therefore, the behavior of CD4^+^ SCs and/or their progeny during wound healing was analysed by inducing full-thickness incisional (incW) and/or excisional wounding (excW) of the EBS and/or ETS of *CD4Cre/tdT* and *CD4CreERT2/tdT* mice. Both wounding protocols resulted in an extensive tdT^+^ labeling of epidermal cells in *CD4Cre/tdT* mice at 13 weeks of age, whereas tdT^+^ epidermal cells are only rarely detected in the unwounded EBS of age-matched *CD4Cre/tdT* mice (Fig. 5A)^12^. 18 days post incW of the EBS, most wound-induced anagen hair follicles were tdT^+^. After 35 days, also wound-near endogen anagen hair follicles as well as BL- and suprabasal IFE cells expressed tdT (Fig. 5A). Likewise, 10- and 17-days post excW of the EBS, tdT expression was observed in hair follicles and in all layers of the IFE in wound-near regions while unwounded EBS of 11 weeks old *CD4Cre/tdT* mice showed no tdT^+^ hair follicles or IFE cells (Fig. 5B). Comparably, 10-, 17- and 51-days post excW of the ETS, tdT was expressed in wide areas of re-epithelized IFE as well as in the IFE and occasionally in hair follicles surrounding the wound edges while unwounded ETS of 15 weeks old *CD4Cre/tdT* mice only rarely showed tdT^+^ IFE (Fig. 5C). tdT^+^ epidermal cells were also detected in the re-epithelized basal layer and suprabasal IFE of the ETS from inducible *CD4CreERT2/tdT* mice that were wounded and simultaneously tamoxifen-treated at an age of 9.5 weeks and analyzed 45 days post excW (Suppl. Fig. S10).

These data show that wound healing-associated processes stimulate the progeny of *CD4Cre* and *CD4CreERT2*-targeted progeny to grow as polygonal units spanning from the healthy skin towards the wound center, as it has been reported for THY1-, K14- or LRIG1-labeled cells^6,20^. Thus, wounding seems to stimulate CD4 expression and self-renewal either of CD4^+^ epidermal cells or of their progeny above age-appropriate levels.

### Loss of CD4 accelerates epidermal wound healing in aged mice

**Fig. 6:**
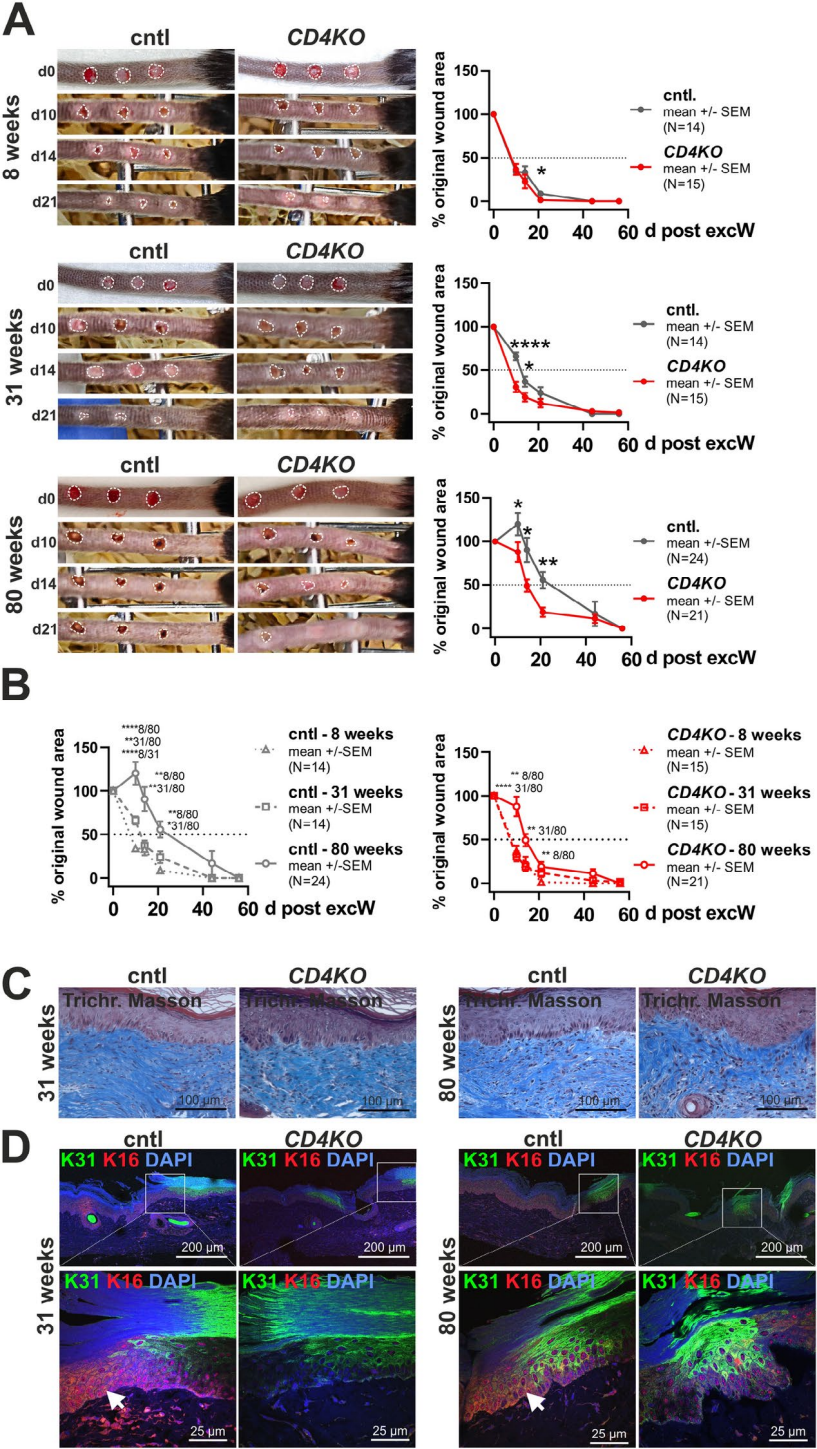
Accelerated wound healing of ETS excW in *CD4KO* mice. Analyses of the EBS after excisional wounding (excW) of 8, 31 and/or 80 weeks-old *CD4KO* and corresponding cntl mice. Wounds of middle-aged and aged *CD4KO* mice heal significantly faster than those of respective cntl mice 10, 14 and/or 21 days after excW (**A**,**B**) without fibrosis or abnormal scarring (**C**) but with decreased K16 expression at the wound edges (**D**). (**A**,**B**) Analysis of the macroscopic healing (A) and the wound sizes of excW from the day of wound induction (d0) up to 56 days post wounding of the ETS of 8, 31 and 80-weeks-old *CD4KO* and age-matched control wt mice (cntl) (A,B). White dotted lines in (A) define the wound area. Per mouse three excW were inflicted. N corresponds to number of wounds. *P < 0.05, ** P < 0.01, **** P < 0.0001. See Suppl. Tables S2 and S9 for animal numbers and means, SEM and P values. (**C**,**D**) Trichrome Masson staining (C) and anti-K31/anti-K16 (D) antibody-stained paraffin sections of wound-near areas 56d post excW of 31 and 80 weeks old *CD4KO* and age-matched cntl. Nuclei were visualized with DAPI. White arrows: K16^+^ IFE. White boxes: zoom-in areas.

CD4 expression in epidermal SCs or the expansion of their progeny is associated with ageing^12^ and wound healing processes, whereas CD4 loss leads to overgrowth of the sc-SCs compartment and INF with age. This suggests that epidermal wound healing might be altered in *CD4KO* mice, particularly with age. Thus, healing, closure and histology of ETS excW of young, middle-aged and aged *CD4KO* and cntl mice were analyzed (Fig. 6A–D).

With increasing age, wound healing is delayed^24,25^. Accordingly, wound healing efficiency in *CD4KO* and cntl mice decreased significantly with age (Fig. 6B). However, while no gross healing differences between young *CD4KO* and cntl mice were observed, middle-aged and aged *CD4KO* mice showed significantly faster wound closure at 10, 14 and/or 44 days after excW compared to age-matched cntl (Fig. 6A,B). Thus, middle-aged and aged *CD4KO* mice showed the healing capacity of young mice and middle-aged cntl mice, respectively.

None of the *CD4KO* or cntl mice showed fibrosis or abnormal scarring (Fig. 6C). The injury-induced protein K16, which is expressed during re-epithelialization at the wound edges^26–28^, was expressed in the wound areas of middle-aged and aged cntl mice, but not or only weak in middle-aged and aged *CD4KO* mice 56 days after excW, respectively (Fig. 6D, Suppl. Fig. S11A). However, irrespective of the smaller scale size, K31 was still expressed at the wound edges of *CD4KO* mice (Fig. 6D). Similarly, KI67, K10 or SCA-1 expression pattern did not differ between the wound areas of middle-aged and aged cntl and *CD4KO* mice (Suppl. Fig. S11A,B).

These data demonstrate that CD4 loss in young mice, despite an increased fc-SCs proliferation (see above), does not affect the wound healing capacity. In aged mice, however, the accumulation of sc-SCs and strong proliferation of INF cells (see above) is accompanied by accelerated wound healing.

## Discussion

By in vivo CD4-lineage tracing and *CD4* knockout we analyzed the role of CD4 in the epidermis and identified it as a factor required for the maintenance of the epidermal SC balance during ageing. Although *CD4KO* mice have been extensively used in immunological research, a skin phenotype has not been described yet. This is probably due to the fact that most studies have used young to middle-aged *CD4KO* mice^15–19^, which are also macroscopically inconspicuous in our hands. Nevertheless, *CD4KO* mice are less prone to skin tumor formation, and it has been hypothesized that this is caused by lack of tumor-promoting CD4 T helper cells^15–18^. However, *CD4KO* keratinocytes isolated from DMBA/TPA-induced lesions are less prone to generate cell lines and show a more benign behavior than cells isolated from corresponding wt cntl^16^. Thus, a cell-intrinsic role of CD4 is very likely.

Indeed, our data show that INF and BL-IFE cells can express CD4, and that their progeny grow as long-term survivors tending to colonize the hair follicles in the EBS and the scale and interscale territory of the IFE in the ETS. These growth preferences match the compartments with the most obvious anomalies in *CD4KO* mice, namely EBS alopecia and altered scale/interscale ETS morphology. Thus, the phenotype of *CD4KO* mice is comparable to that of mice deficient in genes involved in maintaining the epidermal SC balance^23,29–34^.

Potentially, the increased SCA-1 expression explains the phenotype of *CD4KO* EBS and ETS. CD4^+^ keratinocytes (a) express SCA-1^11^, (b) like SCA-1^+^ cells^35^ reside in the BL-IFE and INF, (c) like SCA-1^+^ SCs of the BL-IFE and INF^36^ contribute to the long-term renewal of all IFE layers, (d) like SCA-1^+^ progeny^35^ their progeny colonize all hair follicle layers and form new hair follicles in in vivo transplantation assays, and (e) similar to mice that overexpress SCA-1 in K14^+^ keratinocytes (*K14Cre/Sca-1*)^37^ confer susceptibility to DMBA/TPA-induced tumors^16,17^. Unfortunately, the exact role of SCA-1 in IFE homeostasis is unknown.

The CD4^+^ SCs population may also overlap with LRIG1^+^ sc-SCs that express low SCA-1 levels and reside in the junctional zone and the lower INF and can generate all hair follicle lineages^23,38^. Like CD4 loss, loss of LRIG1 leads to an increased IFE proliferation and altered scale/interscale morphology, but contrary to CD4^+^ cells, LRIG1^+^ SCs only contribute to the IFE under wound healing conditions^23^. In addition, the morphological changes of the *CD4KO* ETS were not accompanied by altered LRIG1 expression or WNT signaling, which are also essential for normal scale/interscale development^21,22^. Thus, CD4 affects the maintenance rather than the development of scale/interscale compartmentation, independently of either LRIG1 or WNT signalling.

The altered scale/interscale morphology of *CD4KO* ETS contrasts normal ageing conditions. Normally, the number of SLC1A3^+^ fc-SCs decreases and SOX6^+^ sc-SCs expand into the fc-SC territory with age^8^. The overlying differentiated layers develop inversely with age, whereas the K31^+^ scale remains unchanged while the K10^+^ interscale areas shrink^9^. However, in the *CD4KO* ETS, the SLC1A3^+^ fc-SCs disappear faster than in age-matched cntl and the overlying K31^+^ scales shrink instead of remaining unchanged. Reduced scale size, as in *CD4KO* mice, is also observed when WNT signaling is inhibited in K14^+^ keratinocytes (*K14/ΔLEF1*). However, in *K14/ΔLEF1* ETS the territory of the fc-SCs is overgrown by strongly proliferating sc-SCs^21^, while in *CD4KO* mice the proliferation of sc-SCs is unchanged. Thus, *CD4* loss rather induces a loss of fc-SCs and K31^+^ cells, whose territory is filled by sc-SCs and the overlying K10^+^ cells. This contrasts aged wt or prematurely aged skin, in which loss of fc-SCs^8,9,22^ is associated with smaller K10^+^ interscales^9^. This suggests decelerated rather than accelerated ageing in *CD4KO* mice. Consistently, aged *CD4KO* mice show better healing capacity than age-matched cntl mice.

During epidermal healing, SCs of the hair follicle and the IFE are interchangeable^5^. This process is mainly driven by INF cells^3,20,39^ and sc-SCs of the IFE^3,4,6^, while fc-SCs rather contribute to the IFE homeostasis^3,4,6^. This is in line with the phenotype observed in *CD4KO* mice. The skin of young *CD4KO* mice shows a high fc-SCs proliferation rate. However, the healing capacity is unchanged compared to age-matched cntl skin. In contrast, the skin of aged *CD4KO* mice show an increased proliferation of INF cells and an accumulation of sc-SCs, which is accompanied by accelerated wound healing. Moreover, in the wt situation, the progeny of CD4^+^ cells are induced in response to wounding and grow in wound-near hair follicles and as typical wound-induced sc-SCs clones from the periphery to the centre of the wound^4,6,20^. Together, all these data support the conclusion that CD4^+^ SCs and their progeny play an essential role in the skin healing process. Beyond that, the observation that *CD4* loss results in SC expansion and alopecia additionally suggests that CD4^+^ cells and their progeny are essential for maintaining SC balance in aged skin.

In fact, we showed that CD4 marks a subpopulation of CD34^+^SCA-1^+^ epidermal cells, whose offspring develop to bulge and BL-IFE cells. We currently do not know whether CD4 acts as a signaling mediator in these cells. In addition, we cannot say whether CD4 loss in *CD4KO* mice leads to a depletion of an epidermal cell population or to an altered signaling that changes their cellular fate. Thus, conditional *CD4KO* or adoptive transfer analyses are needed to provide a more specific picture of the role of CD4 in keratinocytes, which is independent of CD4-depleted immune cells (e.g. CD4^+^ T cells, macrophages, Langerhans’ cells). Unfortunately, *CD4*^*flox*^ mice do not exist, and adoptive transfer requires time-consuming approval processes. To circumvent this methodological limitation, we performed analyses on in vitro cultured keratinocytes isolated from different aged *CD4KO* mice. In line with the results obtained of our in vivo analyses, these experiments showed that despite the absence of immune cells the SC capacity of epidermal SCs from middle-aged *CD4KO* mice is better than age-matched cntl SCs. In addition, depletion of CD4^+^ immune cells has been never connected to imbalanced SC homeostasis in the skin, but rather results in inflammatory skin disease (e.g. in DiGeorge syndrome^40^), decreased susceptibility to skin tumors (e.g. *CD4KO* mice^15–17^) or increased susceptibility to papillomavirus-induced warts (e.g. human CD4 deficiency^41–43^). Thus, the analyses shown here strongly indicate that CD4 expression is essential for the maintenance SC balance in ageing skin, while simultaneously counteracting wound healing. It remains to be seen, which cellular and molecular interaction partners mediate the function of CD4 in the skin and whether the murine data shown here can be transferred to human skin.

## Experimental procedures

### Mice

The study was approved by the Lower Saxony State Office for Consumer Protection and Food Safety (file numbers 33.9-42502-04-15/1926; 33.9-42502-04-11/0374; 33.11.42502-04-20/3535).

All animal experiments were performed in compliance with ARRIVE guidelines and German legal and ethical requirements.

We confirm that all methods were performed in accordance with the relevant guidelines and regulations.

The following mouse strains were used: C57Bl6/N, *Tg(Cd4-cre)1Cwi/Bflu* (*CD4Cre*, JAX stock #017336)^44–46^, *Tg(Cd4-cre/ERT2)11Gnri* (*CD4CreERT2*, JAX stock #022356)^47^, *Gt(ROSA)26Sor*^*tm9(CAG-tdTomato)Hze*^ (*tdT,* JAX stock #007905)^48^ and *B6.129S2-Cd4*^*tm1Mak*^*/J* (*CD4KO*, JAX stock #002663)^14^, *Foxn1*^*nu/nu*^ (JAX stock # 002019). All used mouse strains were maintained on C57BL/6 background. Genotyping was conducted by PCR on genomic DNA isolated from tail or ear clips using primer pairs recommended by The Jackson Laboratory. Isoflurane or CO_2_ anesthetized mice were sacrificed by cervical dislocation. Both genders of transgenic mice were used. No sex-specific differences were observed. Numbers of analyzed *CD4KO* and corresponding cntl mice are given in Supplemental Tables S1 and S2.

*CD4ERT2/tdT* mice were intraperitoneally injected with 1 mg tamoxifen in sunflower oil per day on 2–7 days at an age of 4.5 or 9.5–11 weeks as indicated in the respective figures. *CD4Cre/tdT* mice were intraperitoneally injected with 50 mg/kg body weight 5-bromo-2ʹ-desoxyuridine (BrdU) at an age of 8 weeks. BrdU incorporation was analyzed 3 days post labelling. incW and 5 (back) or 3 mm (tail) diameter excW were inflicted on the shaved telogen back or tail skin^11,20,49^. Wound closure was monitored for up to 56 days post wounding by measuring the wound size using a digital caliper and by photo documentation. Intradermal transplantation of keratinocytes labelled with 5 µM CellTracker™ Green CMFDA Dye (Invitrogen, Germany) into *Foxn1*^*nu/nu*^ mice was performed as previously described^50^.

### Isolation of ETS sheets, individual hair follicles, EBS and ETS cells

Isolation of ETS sheets^51^, individual hair follicles of EBS^52^, EBS^11^ and ETS cells^53^ were performed as described in the respective literature.

### Tissue embedding and sections

Tissue samples were fixed in 4% paraformaldehyde/1× PBS at 4 °C for 2–3 days and either dehydrated and embedded in paraffin or equilibrated overnight in 20% sucrose/1× PBS at 4 °C and embedded in cryo medium (Medite, Germany). Samples were sectioned using a microtome or a cryotome and used for antibody or for hematoxylin/eosin staining, Oil red or Masson Trichrome staining.

### Primary keratinocyte culture

300,000 or 2500 freshly isolated telogen EBS cells were cultured in triplicates under feeder-free conditions on 1:1-diluted Cultrex Basement Membrane Extract (BMA, R&D Systems, USA)/advanced DMEM/F12 (Thermo Fisher Scientific Inc., USA)-coated wells of 6-well plates or in 10 µl BMA droplets (3D) for 7 days in epidermal expansion medium for specific expansion of keratinocytes^36,54^. For Western blot analysis 150,000 p0 keratinocytes/well of 6-well plates were subcultured for 48 h and subsequently harvested in RIPA buffer^55,56^. Organoid number and size were quantified 7 days post seeding of p0 keratinocytes by brightfield microscopy followed by manually counting and by outlining the organoids using the magnetic tool of the Adobe Photoshop CS5 software, respectively.

### Immunostaining, Western blot, flowcytometric, FACS and microscopy analyses

Immunostainings of paraffin and cryotome-sections and ETS sheets as well as flow cytometric analyses, fluorescent activated cell sorting (FACS) of keratinocytes and Western blot analyses of snap frozen skin samples or cultured cells were described previously^11,12,55–57^.

Immunostainings, histological stainings or whole mount analyses were documented on a confocal laser scanning microscope equipped with Fluoview software (Olympus Corporation, Japan), BX60 transmitted-light microscope equipped with Cellsense software (Olympus Corporation, Japan), a fluorescent dissecting microscope (Leica M205FA) equipped with a digital DFC camera (Leica DFC 450C) and the software Leica Application Suite, respectively. The number of hair follicles, length of the scales, thickness of EBS and ETS IFE (between the basal lamina and the lower border of the *stratum corneum*) as well as infundibula length were determined on anti-K10 or anti-K31 antibody or H&E-stained paraffin sections using the measuring tool of the ImageJ software (National Institutes of Health, USA). KI67^+^ cells of the infundibula and the BL-IFE of K31^+^ scales and K10^+^ interscales were counted on anti-KI67/anti-K10 or anti-K31 antibody-stained paraffin sections using the counting tool of the Photoshop CS5 software (Adobe Systems Inc., USA).

35 out of 144 hair follicles randomly isolated from the EBS of a 43 weeks-old *CD4Cre/tdT* mouse contained tdT^+^ cells (~ 24%). 5 tdT^+^ individual hair follicles were excluded due to damage. The remaining were used for determination of hair, clone and lineage type as previously described^52,58–61^.

For Western blot analysis the samples were run on NuPage 4–12% Bis–Tris Midi gels in MES running buffer (Invitrogen, Germany) and semidry blotted onto nitrocellulose membranes^55,56^. SeeBlue Plus2 Prestained Standard (Invitrogen, Germany) for determination of protein sizes was included on each gel/blot. Protein extract from C67BL6/N thymus was simultaneously analyzed as a positive control for CD4 expression and as a negative control for keratinocytes marker expression. Western blot detection was performed using ECL™ Prime Western Blotting Detection Reagents (Merck KGaA, Germany) and the c300 System (Azure Biosystems, USA). The original blots and ECL detection are presented in Supplemental Figures. S12–15.

Flow cytometric analyses or FACsorting were conducted on LSR II flow cytometer (BD Biosciences Pharmingen, USA) or BD ARIA II FACS (BD Biosciences Pharmingen, USA), respectively. Data acquisition and analysis were performed using BD FacsDiva (BD Biosciences Pharmingen, USA) and FlowJo (Treestar, USA) software.

Used sectioning method, retrieval method, antibodies and antibody concentrations are summarized in Supplemental Tables S10–12.

### Transcriptome analyses

For transcriptome analyses, epidermal cells were isolated from 29 to 32-weeks old male *CD4Cre/tdT* mice, stained with CD49f-PerCP-Cy5.5, CD34-FITC and Sca-1-PE-Cy7 antibodies. tdT^neg^CD49f^+^CD34^+^SCA-1^+^, tdT^+^CD49f^+^CD34^+^SCA-1^+^, tdT^neg^CD49f^+^CD34^+^ SCA-1^neg^ and tdT^+^CD49f^+^CD34^+^SCA-1^neg^ cells were sorted by FACS. In total, three biological replicates (each consisting of material from two male mice) were analysed. RNA was retrieved by resuspension/homogenization of 5 × 10^5^ cells in 360 µl TRIzol^®^ Reagent (Life Technologies Co., USA) for 5 min at RT. After chloroform addition, samples were vortexed, centrifuged (12,000×*g*, 15 min, 4 °C) and RNA was precipitated from the aqueous phase by 70% ethanol addition and overnight incubation at -20 °C. Next, RNA was pelleted (12,000×*g*, 45–55 min, 4 °C), washed with 70% ethanol, dried and solved in RNase free water (Invitrogen, USA). RNA quality control (Fragment Analyzer, Agilent Technologies, USA), cDNA library preparation (TruSeq^®^ RNA Sample Preparation v2; Illumina, USA) and RNA sequencing (HiSeq 4000; Illumina, USA) were performed at the NGS Service Facility for Integrative Genomics (NIG), University Medical Center Göttingen, Germany. Raw read & Quality check: Sequence images were transformed with Illumina software BaseCaller to BCL files, which was demultiplexed to fastq files with bcl2fastq v2.20.0.422. The sequencing quality was asserted using FastQC (http://www.bioinformatics.babraham.ac.uk/projects/fastqc/) (version 0.11.5). Mapping & Normalization: Sequences were aligned to the reference genome of M. musculus (mm10 version 96, https://www.ensembl.org/Mus_musculus/Info/Index) using the STAR aligner(Dobin et al., 2013) (version 2.7.1a) allowing for 2 mismatches within 50 bases. Read counting was performed using featureCounts (Liao et al., 2014) (version 1.5.0-p1). Read counts were analyzed in the R/Bioconductor environment (version 3.4.4, http://www.bioconductor.org) using the DESeq2 (Love et al., 2014) package version 1.18.1. Candidate genes were filtered using an absolute log2 fold-change > 1 and FDR-corrected p-value < 0.05. Genes were annotated using the Mus Musculus gene version 96 Gene transfer format (GTF) file. Final data visualization was performed with RStudio (RStudio, Inc., USA).

### Graphs and statistics

Graphs and statistics were conducted using the GraphPad Prism 10 software. Significance was tested by Fisher’s exact test (Fig. 1B) or by non-parametric Mann–Whitney method.

## Supplementary Information

Supplementary Information.

## Data Availability

The datasets used and/or analyzed during the current study are available from the corresponding author on reasonable request. This study did not generate new unique reagents. The accession number for the sequencing data reported in this paper is Gene Expression Omnibus (GEO): GSE266403.
