# Supplementary material for: CD4 expression controls epidermal stem cell balance
Source: Sci Rep. 2025 Feb 4;15:4185. doi: 10.1038/s41598-025-87915-7 (PMC11794708; doi:10.1038/s41598-025-87915-7)
Supplement: Supplementary file 1 — Supplementary Information. [file 41598_2025_87915_MOESM1_ESM.pdf]

Supplemental material

CD4 Expression Controls Epidermal Stem Cell Balance

Nadine Brandes, Heidi Hahn, Anja Uhmann

Supplemental Material

Supplemental Table S1: Number of mice used for back skin analyses shown in Figure 1 and Supplemental Figure S2.

	age of the mice	Fig. 1B	Fig. 1D	Fig. 1G	Fig. 1H	Fig. 1H	Suppl. Fig. S2B,C,F
**cntl**	**8 weeks**	11	7	n.a.	3	3	2
CD4KO		7	7	n.a.	3	3	2
**cntl**	**31 weeks**	12	4	n.a.	4	4	3
CD4KO		12	4	n.a.	4	4	3
**cntl**	**80 weeks**	11	6	9	4	4	n.a.
CD4KO		14	7	5	4	4	n.a.

Supplemental Table S2: Number of mice used for tail skin analyses shown in (Supplemental) Figures 2, S3, S4 and 6.

	age of the mice	Fig. 2C	Suppl. Fig. S2C	Suppl. Fig. S3B	Fig. 2D	Fig. 2E	Fig. 2F	Suppl. Fig. S4F	Suppl. Fig. S4G	Suppl. Fig. S4H	Suppl. Fig. S4I	Fig. 6
**cntl**	**8 weeks**	3	2	n.a.	3	3	3	3	2	2	3	5
CD4KO		5	3	n.a.	3	3	3	3	3	3	3	5
**cntl**	**31 weeks**	4	4	n.a.	3	3	3	3	4	4	3	5
CD4KO		3	4	n.a.	2	2	2	2	4	4	2	5
**cntl**	**80 weeks**	6	4	5	6	6	6	6	4	4	6	8
CD4KO		6	4	5	7	7	7	7	4	4	7	7

Supplemental Table S3: Number and percentage of *CD4KO* and cntl mice with and without back skin fur abnormalities.

	**cntl**		***CD4KO***				
**age of mice**	**no fur abnormalities**	**fur abnormalities**	**no fur abnormalities**	**all fur abnormalities**	**thinning back skin fur**	**partial back skin alopecia**	**back skin alopecia**
**8-10 weeks**	11 / 100%	0 / 0%	7 / 100%	0 / 0%	0 / 0%	0 / 0%	0 / 0%
**31-33 weeks**	12 / 100%	0 / 0%	9 / 75%	3 / 25%	0 / 0%	3 / 25%	0 / 0%
**67-80 weeks**	11 / 100%	0 / 0%	2 / 14.3%	12 / 85.7%	4 / 28.6%	2 / 14.3%	6 / 42.9%

Supplemental Table S4: Number of wild-type mice used as donors for flow cytometric analyses of the CD4 expression of EBS cells shown in Supplemental Figure S5C,D.

age of the mice	Suppl. Fig. S5C,D
**5 weeks**	3
**46 weeks**	2
**55 weeks**	2

Supplemental Table S5: Mean ± SEM and P values (Fig. 1B: Fisher's exact test; Fig. 1D,E,G,H Mann-Whitney method) of the graphs shown in Figure 1.

mice	age of the mice	back skin abnormalities Figure 1B		number of HF/4.8 mm skin Figure 1D		IFE thickness [µm] Figure 1H		infundibulum length [µm] Figure 1H	
		%	P values	mean ± SEM	P values	mean ± SEM	P values	mean ± SEM	P values
**cntl**	8 weeks	0%	>0.9999	7.674 ± 0.267	0.0275	12.48 ± 0.4369	<0.0001	97.92 ± 2.524	0.0825
CD4KO		0%		8.510 ± 0.282		17.44 ± 0.3983		105.6 ± 2.403	
**cntl**	31 weeks	0%	0.2174	8.148 ± 0.249	<0.0001	14.07 ± 0.4341	0.0006	80.37 ± 3.205	0.0022
CD4KO		25%		5.704 ± 0.164		16.14 ± 0.3991		93.26 ± 2.124	
**cntl**	80 weeks	0%	<0.0001	7.020 ± 0.217	0.0005	15.82 ± 0.3054	0.0001	62.78 ± 1.840	<0.0001
CD4KO		85.71%		5.813 ± 0.243		17.88 ± 0.5054		85.81 ± 3.393	

mice	age of the mice	% KI67+ BL-IFE cells Figure 1G		% KI67+ infundibulum cells Figure 1G	
**cntl**	80 weeks	28.41 ± 1.563	<0.0001	30.63 ± 1.937	<0.0001
CD4KO		59.8 ± 2.872		63.89 ± 3.463	

Supplemental Table S6: Mean ± SEM and P values of the graphs shown in Supplemental Figure S2.

mice	age of the mice	organoid number Suppl. Figure S2B			organoid size [µm^2] Suppl. Figure S2C		
		mean ± SEM	P values		mean ± SEM	P values	
**cntl**	8 weeks	81.33 ± 11.7	0.2606	**cntl 8/31 weeks** 0.0014 ***CD4KO* 8/31 weeks** 0.9156	55.65 ± 1.739	0.1238	**cntl 8/31 weeks** <0.0001 ***CD4KO* 8/31 weeks** <0.0001
CD4KO		67.8 ± 10.45			70.47 ± 3.169		
**cntl**	31 weeks	38.11 ± 5.081	0.0295		107.0 ± 4.981	<0.0001	
CD4KO		66.5 ± 9.793			55.76 ± 2.201		

Supplemental Table S7: Mean ± SEM and P values (Mann-Whitney method) of the graphs shown in Figure 2 and Supplemental Figure S4.

mice	age of the mice	scale length [µm] Figure 2C		scale thickness [µm] Suppl. Figure S4C		interscale thickness [µm] Suppl. Figure S4G		infundibulum length [µm] Suppl. Figure S4H	
		**mean ± SEM**	**P values**	**mean ± SEM**	**P values**	**mean ± SEM**	**P values**	**mean ± SEM**	**P values**
**cntl**	8 weeks	187.7 ± 6.084	0.0035	30.1 ± 0.7115	<0.0001	28.28 ± 0.7518	<0.0001	89.31 ± 2.299	<0.0001
CD4KO		162.6 ± 5.412		37.83 ± 1.331		36.66 ± 0.990		105.6 ± 1.680	
**cntl**	31 weeks	216.0 ± 5.767	<0.0001	37.42 ± 0.7441	06922	33.64 ± 0.5720	0.2516	102.1± 3.133	<0.0001
CD4KO		126.4 ± 7.094		38.36 ± 0.8434		32.87 ± 0.5295		145.4± 3.466	
**cntl**	80 weeks	174.6 ± 4.489	<0.0001	32.26 ± 0.6104	<0.0001	32.45 ± 0.5295	0.0051	156.0 ± 2.906	<0.0001
CD4KO		126.3 ± 5.988		38.89 ± 1.032		34.5± 0.6119		182.8 ± 4.499	

mice	age of the mice	% scale BL-IFE cells Figure 2D		% interscale BL-IFE cells Figure 2E	
**cntl**	8 weeks	35.42 ± 2.822	0.2844	64.58 ± 2.822	0.2844
CD4KO		39.74 ± 1.708		60.26 ± 1.708	
**cntl**	31 weeks	38.84 ± 3.159	0.0041	61.16 ± 3.159	0.0041
CD4KO		24.73 ± 2.986		75.27 ± 2.986	
**cntl**	80 weeks	44.27 ± 2.319	<0.0001	55.73 ± 2.319	<0.0001
CD4KO		16.2 ± 3.329		83.8 ± 3.329	

mice	age of the mice	% scale KI67$^+$ BL-IFE cells Figure 2F		% interscale KI67$^+$ BL-IFE cells Suppl. Figure S4F		% infundibulum KI67$^+$ cells Suppl. Figure S4I	
**cntl**	8 weeks	8.017 ± 1.077	0.0284	12.97 ± 1.61	0.7125	25.68 ± 4.281	0.2910
CD4KO		13.22 ± 1.723		14.71 ± 2.201		33.2 ± 4.168	
**cntl**	31 weeks	9.763 ± 1.034	0.1879	16.14 ± 1.550	0.1153	22.18 ± 2.587	<0.0001
CD4KO		6.996 ± 1.284		22.75 ± 2.999		50.25 ± 4.33	
**cntl**	80 weeks	14.49 ± 1.478	<0.0001	19.51 ± 1.725	0.1095	27.1 ± 2.970	0.0056
CD4KO		4.444 ± 1.093		24.34 ± 2.082		34.74 ± 1.884	

Supplemental Table S8: Mean ± SEM and P values (Mann-Whitney method) of the graphs shown in Supplemental Figure 5C,D.

mice	age of the mice	% CD49f⁺ CD4⁺ EBS cells<br>Suppl. Figure S5C		% CD34⁺ SCA-1⁺ cells of CD49f⁺ CD4⁺ EBS cells<br>Suppl. Figure S5D		% bulge cells of CD49f⁺ CD4⁺ EBS cells<br>Suppl. Figure S5D		% BL-IFE cells of CD49f⁺ CD4⁺ EBS cells<br>Suppl. Figure S5D	
		**mean ± SEM**	**P values**	**mean ± SEM**	**P values**	**mean ± SEM**	**P values**	**mean ± SEM**	**P values**
**wild-type**	5 weeks	0.543 ± 0.288	0.057143	85.5 ± 2.363	0.628571	2.697 ± 0.503	0.057143	0.263 ± 0.122	0.857143
	46-55 weeks	1.453 ± 0.145		83.25 ± 2.001		10.585 ± 1.480		0.172 ± 0.078	

Supplemental Table S9: Mean ± SEM and P values (Mann-Whitney method) of the graphs shown in Figure 6A,B. excW: excision wounding

mice	days post excW	% original wound area (8 weeks-old mice)		% original wound area (31 weeks-old mice)		% original wound area (80 weeks-old mice)			
		mean ± SEM	P values	mean ± SEM	P values	mean ± SEM	P values	P values	
**cntl**	10	33.87 ± 2.87	0.7379	66.09 ± 4.69	<0.0001	120.02 ± 12.96	0.0553	8/80<br>31/80<br>8/31	<0.0001<br>0.0018<br><0.0001
CD4KO		36.49 ± 6.39		30.66 ±5.65		87.82 ± 11.28		8/80<br>31/80<br>8/31	0.0017<br><0.0001<br>0.7099
**cntl**	14	33.07 ± 7.13	0.1881	36.93 ± 5.87	0.0272	90.38 ± 13.93	0.0482	8/80<br>31/80<br>8/31	0.0060<br>0.0078<br>0.7159
CD4KO		22.55 ± 7.92		18.98 ± 4.97		49.15 ± 7.03		8/80<br>31/80<br>8/31	0.0549<br>0.0031<br>0.8008
**cntl**	21	8.77 ± 3.13	0.0123	23.74 ± 6.78	0.1820	55.46 ± 9,46	0.0037	8/80<br>31/80<br>8/31	0.0012<br>0.0164<br>0.1563
CD4KO		1.26 ± 1.26		11.95 ± 4.78		18.69 ± 5.45		8/80<br>31/80<br>8/31	0.0062<br>0.3.91<br>0.0729
**cntl**	44	0	-	0	-	16.67 ± 14.23	-	-	-
CD4KO		0		2.93 ± 2.93		11.17 ± 5.07		-	-
**cntl**	56	0	-	0	-	0	-	-	-
CD4KO		0		1.46 ± 1.46		0		-	-

Supplemental Table S10: Primary and secondary antibodies used for immunofluorescent stainings of cytospun cells, *in vitro* cultured cells, ETS sheets, paraffine or cryostat (cryo) sections.

Antigen	Antibody	Reactivity	Manufacturer (Clone)	Sample Type (Antigen Retrieval)	Dilution
**AE13**	ms anti h/ms	h/ms	Thermo Fisher Scientific, USA (AE13)	paraffine (boil)	1:1,000
**BrdU**	r anti BrdU	-	Abcam, UK (BU1/75)	paraffine (boil)	1:100
**β-CATENIN**	rb anti h	h/ms	Abcam, UK (E247)	paraffine (boil)	1.200
**CD34**	r anti ms	ms	Bio-Rad AbD Serotec GmbH, Germany (MEC14.7)	paraffine (58°C)	1:200
**CD4**	ms anti h	h/ms	Thermo Fisher Scientific, USA (4B12)	cytospun/cryo	1:20
**CD45**	rb anti ms	h/ms	Abcam, UK (polyclonal)	paraffine (boil)	1:150
**GATA3**	ms anti h	h/ms	SantaCruz Biotechnologies, USA (HG3-31)	paraffine (boil)	1:50
**K5**	rb anti-K5	h/ms	BioLegend, Inc., USA (Poly19055)	sheets/cytospun/*in vitro*/ cryo/paraffine (boil/58°C)	1:1,000
**K10**	rb anti ms	h/ms	BioLegend, Inc., USA (Poly19054)	sheets/paraffine (boil/58°C)	1:500
**K16**	rb anti h	h/ms	LifeSpan BioSciences, Inc, USA	paraffine (boil)	1:500
**K31**	gp anti h	h/ms	PROGEN Biotechnik GmbH, Germany	sheets/paraffine (boil)	1:200
**KI67**	ms anti h	h/ms	BD Pharmingen, USA (B56)	paraffine (boil)	1:50
**Langerin**	ms anti h	h/ms	Biotechne, USA (306G9)	paraffine (boil)	1:50
**LEF1**	rb anti	h/ms	Cell Signaling Technology, Inc., USA (C12A5)	paraffine (boil)	1:200
**LRIG1**	gt anti ms	ms	R&D Systems, Inc., USA	sheets/paraffine (58°C)	1:100
**MelanA**	rb anti h	h/ms	Novus Biologicals, USA (A19-P)	paraffine (boil)	1:200
**RFP ***	gt anti-RFP	-	My BioSource, USA (MBS448122)	paraffine (boil)	1:200
**RFP ***	rb anti-RFP	-	Rockland Immunochemicals, Inc., USA (600-401-379S)	paraffine (boil/58°C)	1:500
**SCA-1**	r anti ms	ms	Abcam, UK (E13 161-7)	*in vitro*/paraffine (58°C)	1:100
**SOX6**	rb anti h	ms	Abcam, UK	paraffine (boil)	1:500
**SOX9**	rb anti h/ms	h/ms	Millipore/Merck KGaA, Germany	paraffine (boil)	~~1:2,00~~
**SLC1A3**	rb anti ms	h/ms	Abcam, UK	paraffine (58°C)	1:200
**TCHH**	ms anti h/ms	h/ms	Thermo Fisher Scientific, USA (AE15)	paraffine (boil)	1:50

* detects tdT; gp: genuine pig; ms: mouse, r: rat; rb: rabbit; gt: goat; d: donkey; bov: bovine. Boil: boiling in citric acid, pH6; 58°C: incubation in citric acid, pH6 at 58°C. The following fluorochrome-labeled secondary antibodies were purchased from Jackson ImmunoResearch Laboratories, Inc., USA and used in 1:200 dilution: d anti-ms-Alexa488 (#715-545-150); d anti-rb-Alexa488 (#711-545-152); d anti-rb-Cy3 (#711-165-152); d anti-rb-AMCA (#711-155-152); d anti-rat-Alexa488 (# 712-545-150); d anti-gp-Alexa488 (#706-545-148); bov anti-gt-Alexa488 (#805-545-150); d anti-gt-Cy3 (#705-165-147).

Supplemental Table S11: Primary and secondary antibodies used for Western blot analyses.

Antigen	Antibody	Reactivity	Manufacturer (Clone)	Dilution
**CD4**	ms anti h	h/ms	Thermo Fisher Scientific, USA (4B12)	1:100
**GAPDH**	ms anti ms	h/ms	Cell Signaling Technology, Inc., USA (D4C6R)	1:1,000
**K5**	rb anti-K5	h/ms	BioLegend, Inc., USA (Poly19055)	1:1,000
**K10**	rb anti ms	h/ms	BioLegend, Inc., USA (Poly19054)	1:1,000
**K14**	ms anti h	h/ms	Abcam, UK (LL002)	1:1,000
**K31**	rb anti h	h/ms	antibodies-online (AA 10-90)	1:1,000
**LRIG1**	gt anti ms	ms	R&D Systems, Inc., USA	1:100
**PCNA**	ms anti ms	h/ms	Cell Signaling Technology, Inc., USA (PC10)	1:1,000
**SCA-1**	r anti ms	ms	Abcam, UK (E13 161-7)	1:1,000
**SLC1A3**	rb anti ms	h/ms	Abcam, UK	1:1,000

ms: mouse, r: rat; rb: rabbit; gt: goat. The respective horse radish peroxidase (HRP)-labeled secondary antibodies were purchased from Jackson ImmunoResearch Laboratories, Inc., USA and used in 1:10,00 dilution: rb anti-ms-HRP (#315-035-003); gt anti-rb-HRP (#111-035-045); d anti-gt-HRP (#705-035-003).

Supplemental Table S12: Antibodies used for flow cytometry.

Antigen	Antibody	Reactivity	Clone
**CD34**	r anti-CD34-FITC	ms	RAM34
**CD34**	r anti-CD34-PE	ms	RAM34
**CD4**	r anti-CD4-FITC	ms	RM4-5
**CD49f**	r anti-CD49f-PerCP-Cy5.5	ms/h	GoH3
**SCA-1**	r anti-SCA-1-PE-Cy7	ms	D7

All antibodies were purchased from BD Bioscience, USA.
ms: mouse, r: rat; h: human.

SUPPLEMENTAL FIGURE S1

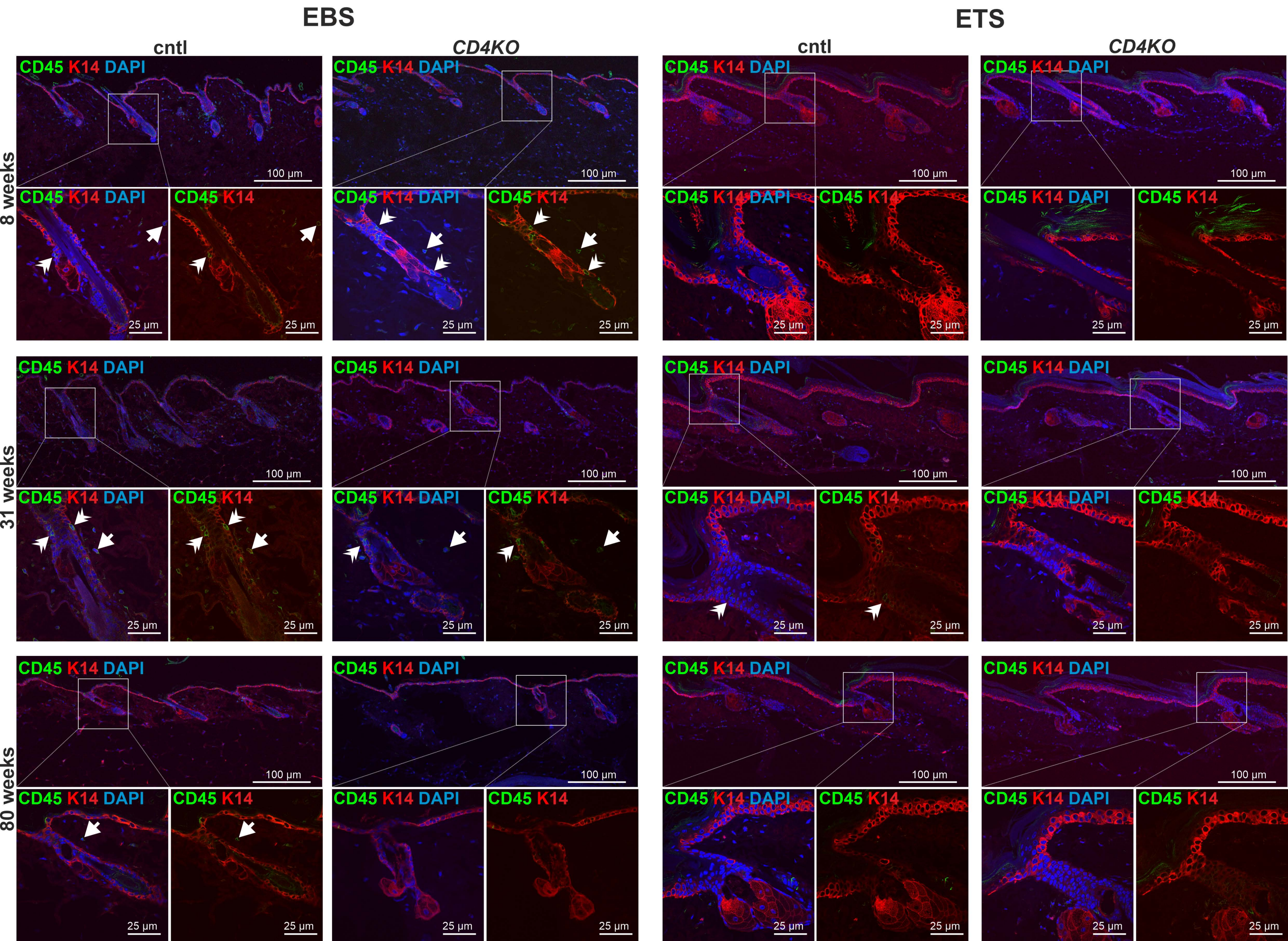

Supplemental Figure S1: Immunofluorescent analyses of CD45-expressing cells in the EBS and ETS of *CD4KO* and corresponding control mice.

Immunofluorescent anti-CD45/anti-K14 stainings of EBS and ETS samples from 8, 31 (A,C-I) and 80 weeks-old *CD4KO* and age-matched control wild-type mice (cntl). Nuclei were visualized with DAPI. White double arrowheads: $CD45^+$ cell in the INF/HF compartment. White arrows: $CD45^+$ cells of the dermis. White boxes: zoom-in area.

SUPPLEMENTAL FIGURE S2

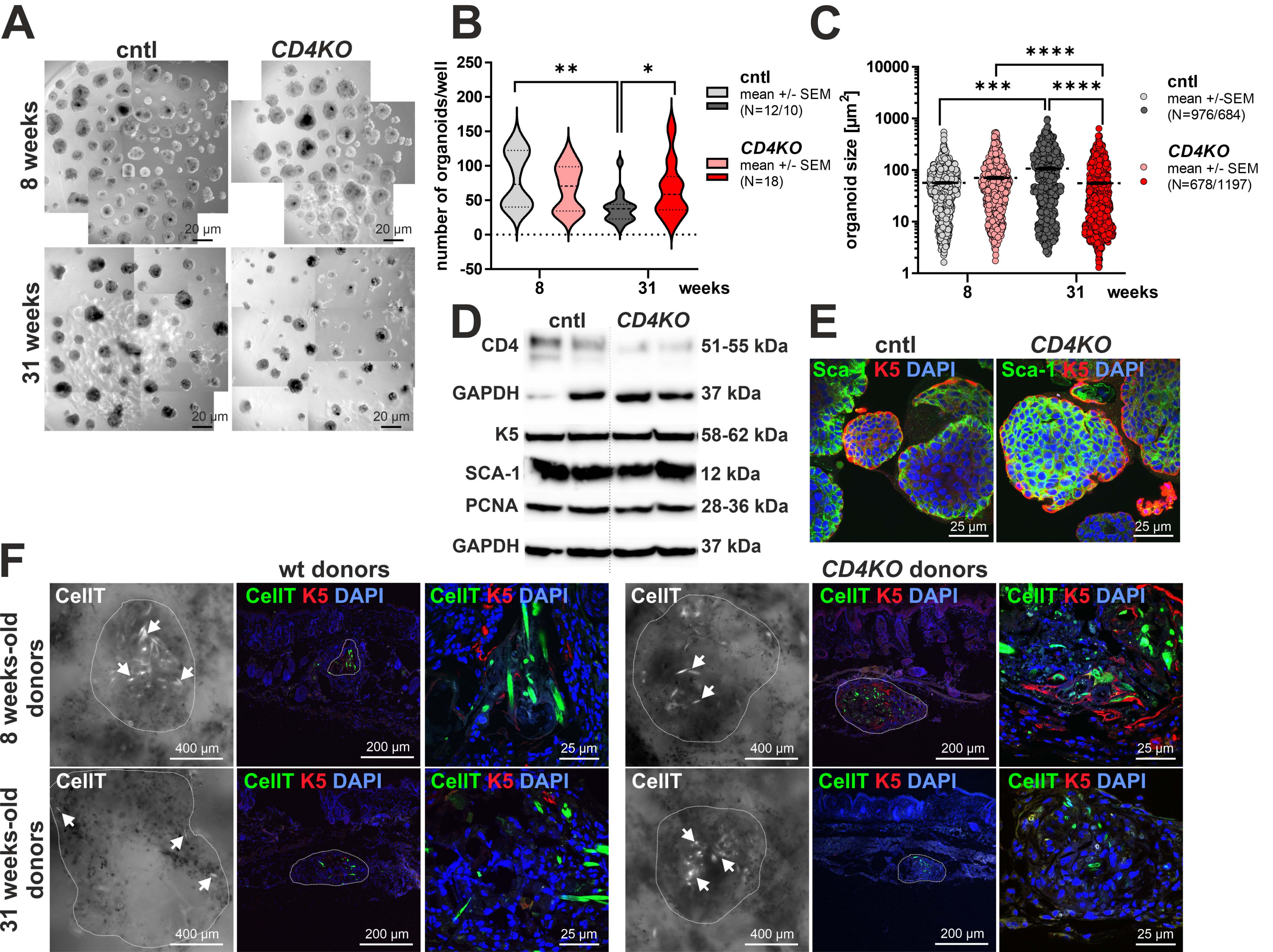

Supplemental Figure S2: ***In vitro*** **and** ***in vivo*** **growth potential of** ***CD4KO*** **EBS cells.**

(A-E) *In vitro* analyses of 3D-cultered (A-C,E) (passage p0) and adherently, feeder-free growing (passage p1) **(D)** EBS cells and **(F)** analysis of *in vivo* SC capacities of EBS cells isolated from 8 (A-C,F) and 31 (A-F) weeks-old *CD4KO* and corresponding control wild-type mice (cntl). Transmitted-light (A), number (B) and size (C) of organoids formed from *CD4KO* and cntl EBS cells. Western blot analyses of cell lysates from adherently, feeder-free growing keratinocytes (D) and SCA-1/ K5 expression of 3D-cultered keratinocytes from 31 week-old donors (E). Fluorescent analyses of CellTracker-labeled *CD4KO* and cntl EBS cells in EBS whole mounts (right) and in anti-K5 antibody stained EBS cryosections (middle and left) of hypodermal transplanted $Foxn1^{nu/nu}$ mice (F). Protein bands in (D) were cropped from original blots shown in Suppl. Fig. S14. Nuclei were visualized with DAPI. Dotted white lines in (F) delimit the transplants. White arrows: $CellTracker^{+}$ HF. (B,C) N correspond to the number of measuring points. * $P < 0.05$, ** $P < 0.01$, ****$P < 0.0001$. See Supplemental Tables S1 and S6 for donor numbers and means, SEM and P values.

SUPPLEMENTAL FIGURE S3

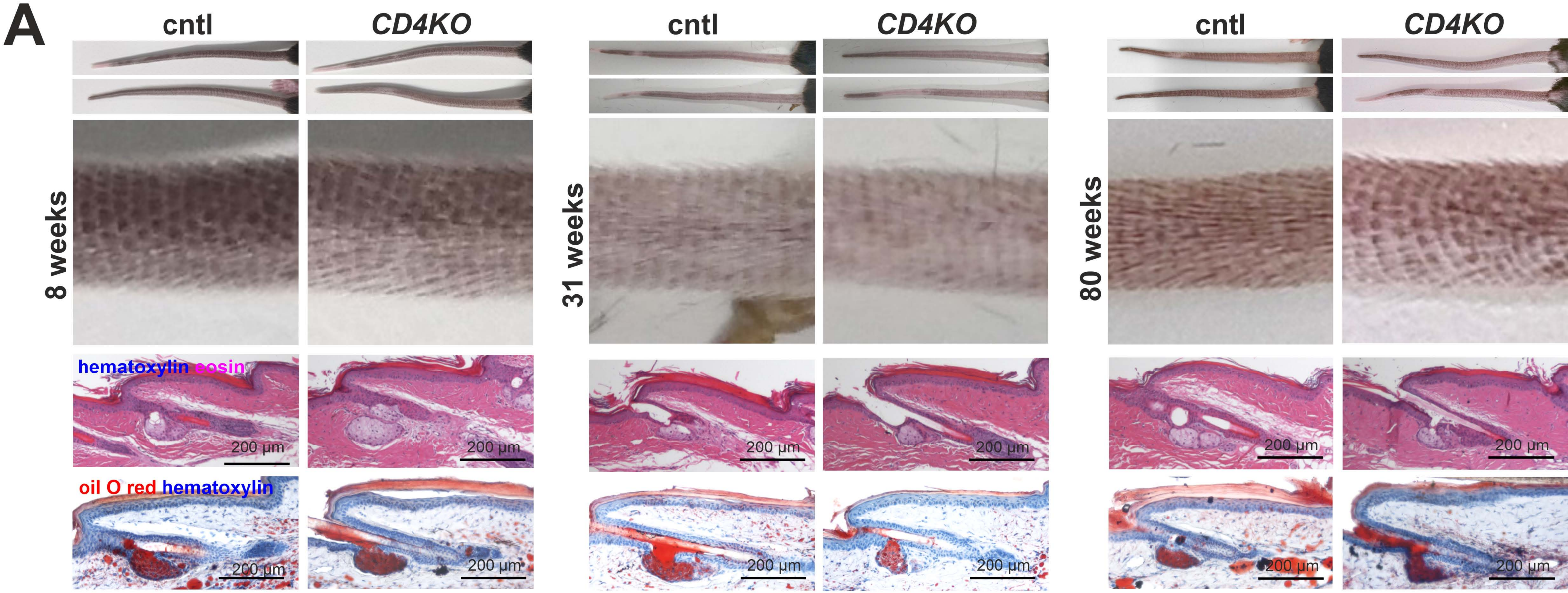

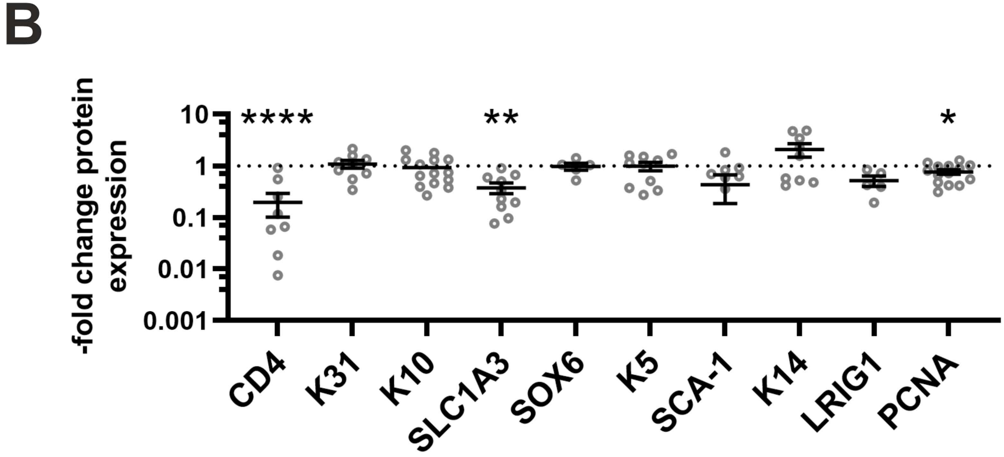

Supplemental Figure S3: The ETS phenotype of *CD4KO* mice.

(A, B) Macroscopic and hematoxylin/eosin and oil O red/hematoxylin (A) and quantification of Western blot-based protein expression analyses (B) of ETS samples from 8 (A), 31 (A) and 80 weeks-old (A,B) *CD4KO* and age-matched control wild-type mice (cntl). Protein expression levels were normalized to the GAPDH expression of the respective sample on the same gel (see Fig. 2). Measuring points in (B) are from analysis of the same samples on two or three Western blots that were processed in parallel. Original blots are presented in Supplemental Figure S15. Expression levels of the cntl were set to 1. * $P < 0.05$, ** $P < 0.01$, **** $P < 0.0001$. See Supplemental Table S2 for animal numbers.

SUPPLEMENTAL FIGURE S4

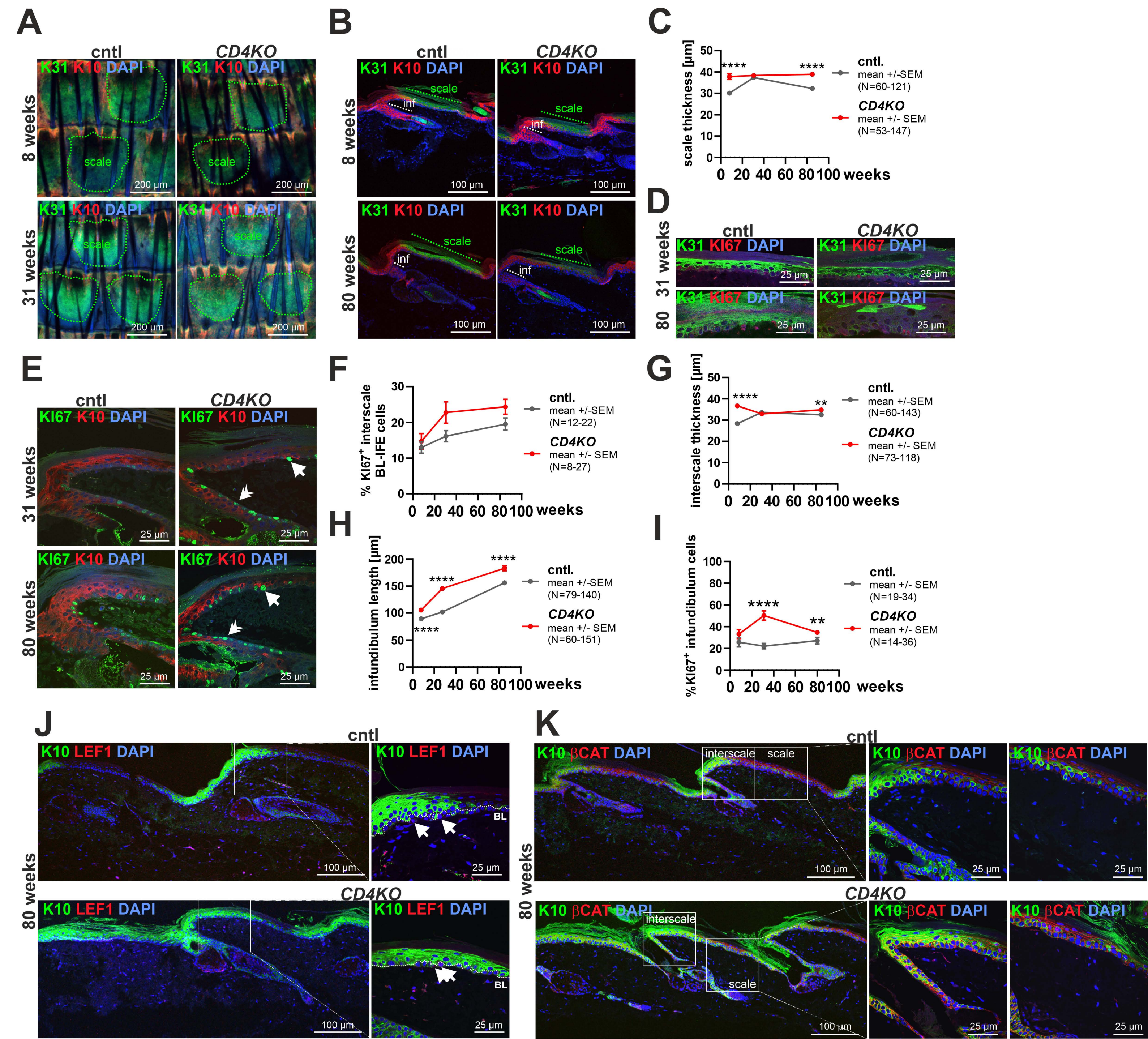

Supplemental Figure S4: The ETS phenotype of *CD4KO* mice.

(A,B,D,E,J,K) Immunofluorescent stainings and **(C,F-I)** quantification of the scale thickness (C), percentage of $KI67^+$ interscale BL-IFE cells (F), interscale thickness (G), infundibulum length (H) and proliferation rate of the infundibula (I) of ETS samples from 8 (A-C, F-I), 31 (A,C-I) and/or 80 weeks-old (B-I,J,K) *CD4KO* and age-matched control wild-type mice (cntl). Anti-K31/anti-K10 (A,B), anti-K31/anti-KI67 (D), anti-KI67/anti-K10 (E), anti-LEF1/anti-K10 (J) and anti-b-CATENIN/anti-K10 (K) antibody-stained ETS sheets (A) or paraffine sections (B,D,E,J,K). Nuclei were visualized with DAPI. White double arrowheads: $KI67^+$infundibulum/HF cells. White arrows: $KI67^+$ BL-IFE or $LEF1^+$ BL-IFE cells. Green dotted lines in (A) delimit the scale borders. Dotted lines in (J) define the basal layer (BL). White boxes: zoom-in areas. Inf: infundibulum. (C,F-I) N correspond to the number of measuring points. ** $P < 0.01$, **** $P < 0.0001$. See Supplemental Tables S2 and S7 for animal numbers and absolute numbers or means, SEM and P values.

SUPPLEMENTAL FIGURE S5

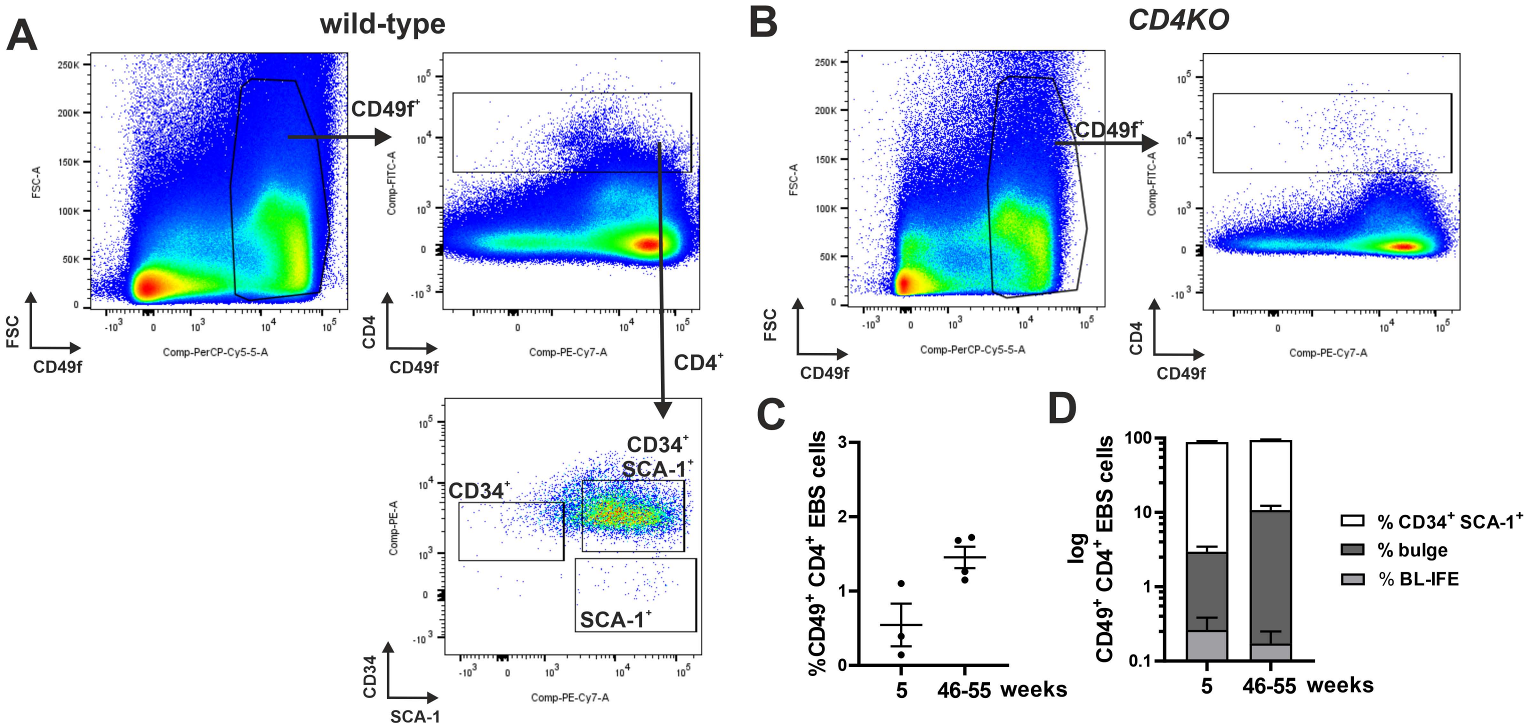

Supplemental Figure S5: Flow cytometric-based quantification of $CD4^+$ keratinocytes in the EBS of young and middle-aged wild-type mice.

(A,B) Representative CD49f/FSC-plotted flow cytometric data of EBS keratinocytes of a 5 weeks-old wild-type mouse. All $CD49f^+$ keratinocytes were plotted against CD4. Subsequently $CD49f^+$ $CD4^+$ keratinocytes of the wild-type mouse were SCA-1/CD34-plotted for determination of the abundancy of $CD4^+$ $CD34^+$ bulge, $CD4^+$ SCA-1^+ BL-IFE and $CD4^+$ $CD34^+$SCA-1^+ epidermal cells (see [1,2]). As a control for the specificity of the used CD4 antibody a similar analysis was performed on EBS cells from a 5 weeks-old *CD4KO* mouse (B). **(C,D)** Percentage of all $CD49f^+$ $CD4^+$ keratinocytes (C) and the fraction of $CD34^+$ SCA-1^+, $CD34^+$ (bulge) and SCA-1^+ (BL-IFE) cells of $CD49f^+$ $CD4^+$ keratinocytes (D) based on flow cytometric analyses as shown in (A) of the EBS of 5 and 46-55 weeks-old wild-type mice. See Supplemental Tables S4 and S8 for animal numbers and absolute numbers or means, SEM and P values.

Supplemental Figure S6

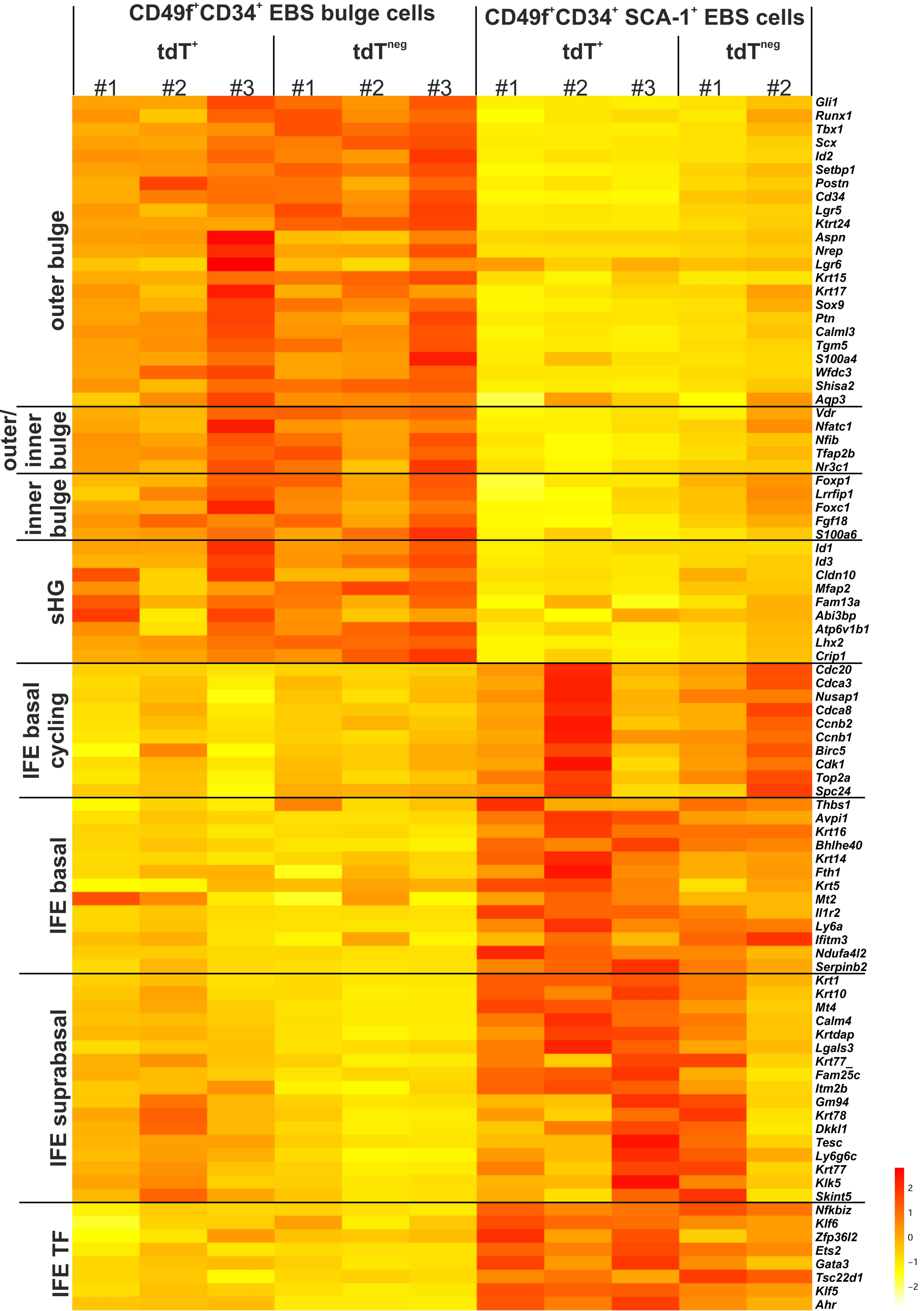

Supplemental Figure S6: The progeny of $CD4^+$ epidermal cells grow as $CD34^+$ bulge and $CD34^+$ SCA-1^+ cells of the EBS.

Transcriptome-based differential gene expression of fluorescence activated cell sorted EBS *CD4Cre/tdT* cells. tdT^+ and tdT^{neg} $CD49f^+CD34^+$ EBS bulge and tdT^+ and tdT^{neg} $CD49f^+CD34^+$ SCA-1^+ EBS cells of 29-31 weeks-old *CD4Cre/tdT* mice were gated as shown in Figure 4A and subsequently sorted. Each biological replicate (#1-3) consists of EBS isolates from 2 male aged-matched mice. Markers of the outer bulge, junctional zone and IFE were chosen based on marker genes of the main epidermal cell populations of telogen epidermis, which had been identified using quantitative single-cell RNAsequencing [3]. sHG, secondary hair germ; TF, transcription factors.

SUPPLEMENTAL FIGURE S7

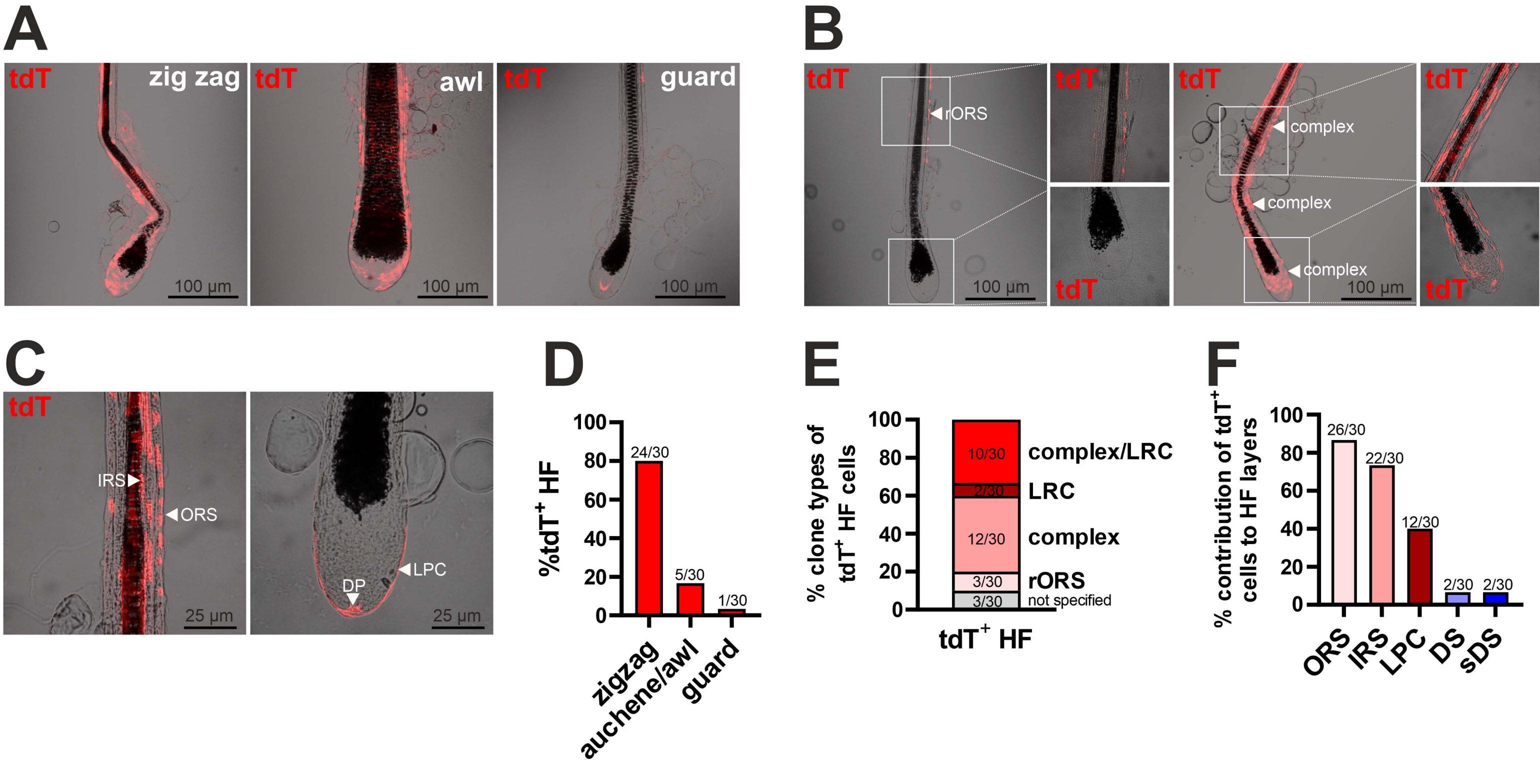

Supplemental Figure S7: The progeny of $CD4^+$ epidermal cells grow in anagen HF of all types, clones and lineages in in the EBS.

(A-C) Representative fluorescent analyses and **(D-F)** quantification of tdT^+ iHF of a 43 weeks-old *CD4Cre/tdT* mouse isolated for determination of the hair type (A,D) and of the clone (B,E) and lineage type (C,F) of 30 HF containing tdT^+ HF cells. DP: dermal papillae; DS: dermal sheath; IRS: inner root sheath; LPC: lower proximal cup; ORS: outer root sheath; rORS: basal ORS clone repopulating the HF from the distal part; sDS: surrounding dermal sheath. Number of analyzed HF are given in the graphs (see also material and method section).

SUPPLEMENTAL FIGURE S8

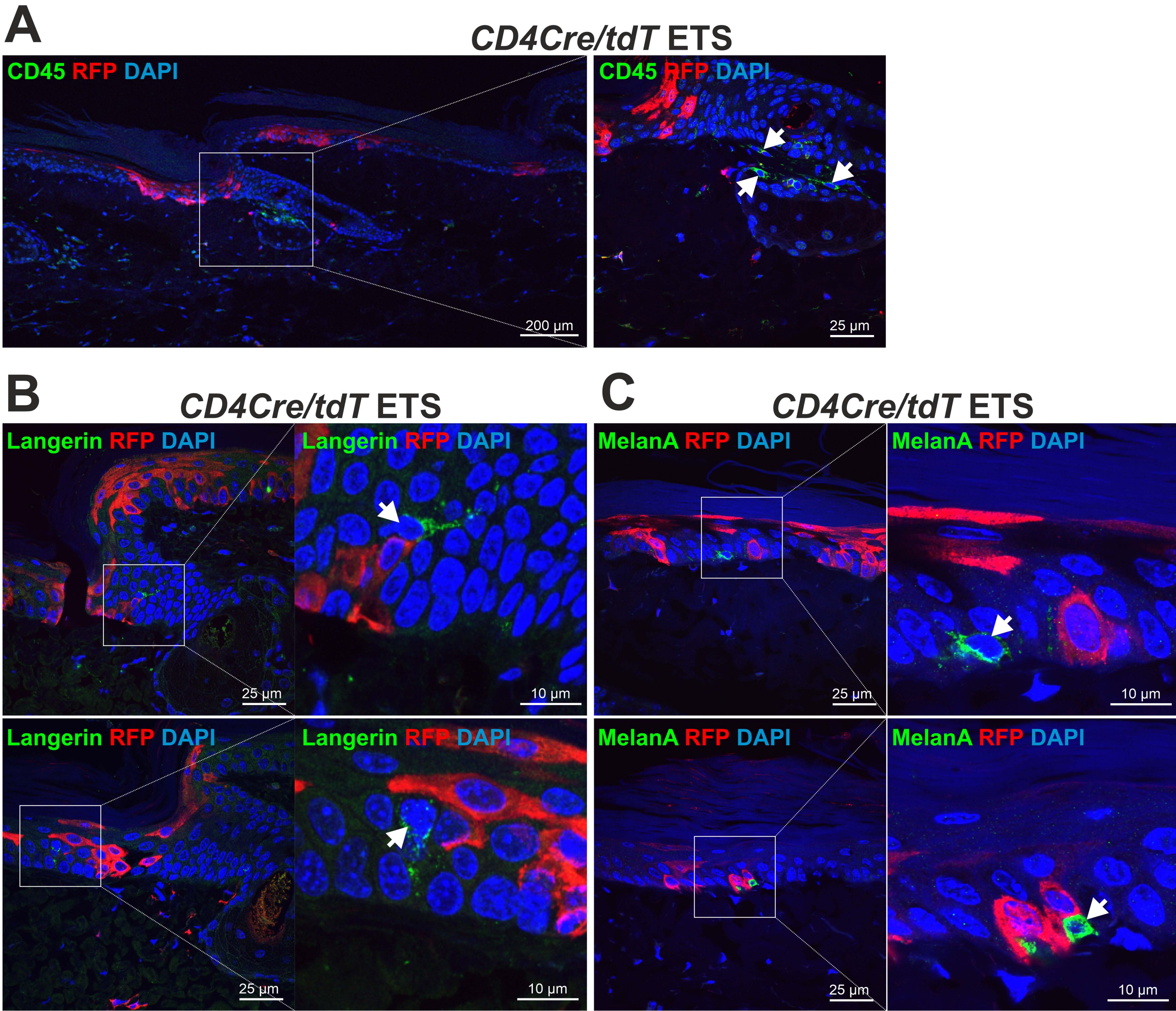

Supplemental Figure S8: The epidermal progeny of $CD4^+$ cells does not express immune cell or melanocyte markers.

(A-C) Fluorescent analyses of ETS paraffin sections of 80 weeks-old mice *CD4Cre/tdT* mice stained with antibodies against RFP for tdT detection and the general immune cell marker CD45 (A), the Langerhans' cell marker Langerin (CD207) (B) or the melanocyte marker MelanA (C). Nuclei were visualized with DAPI. White arrows: $CD45^+$, $Langerin^+$ or $MelanA^+$ cells. White boxes: zoom-in area.

SUPPLEMENTAL FIGURE S9

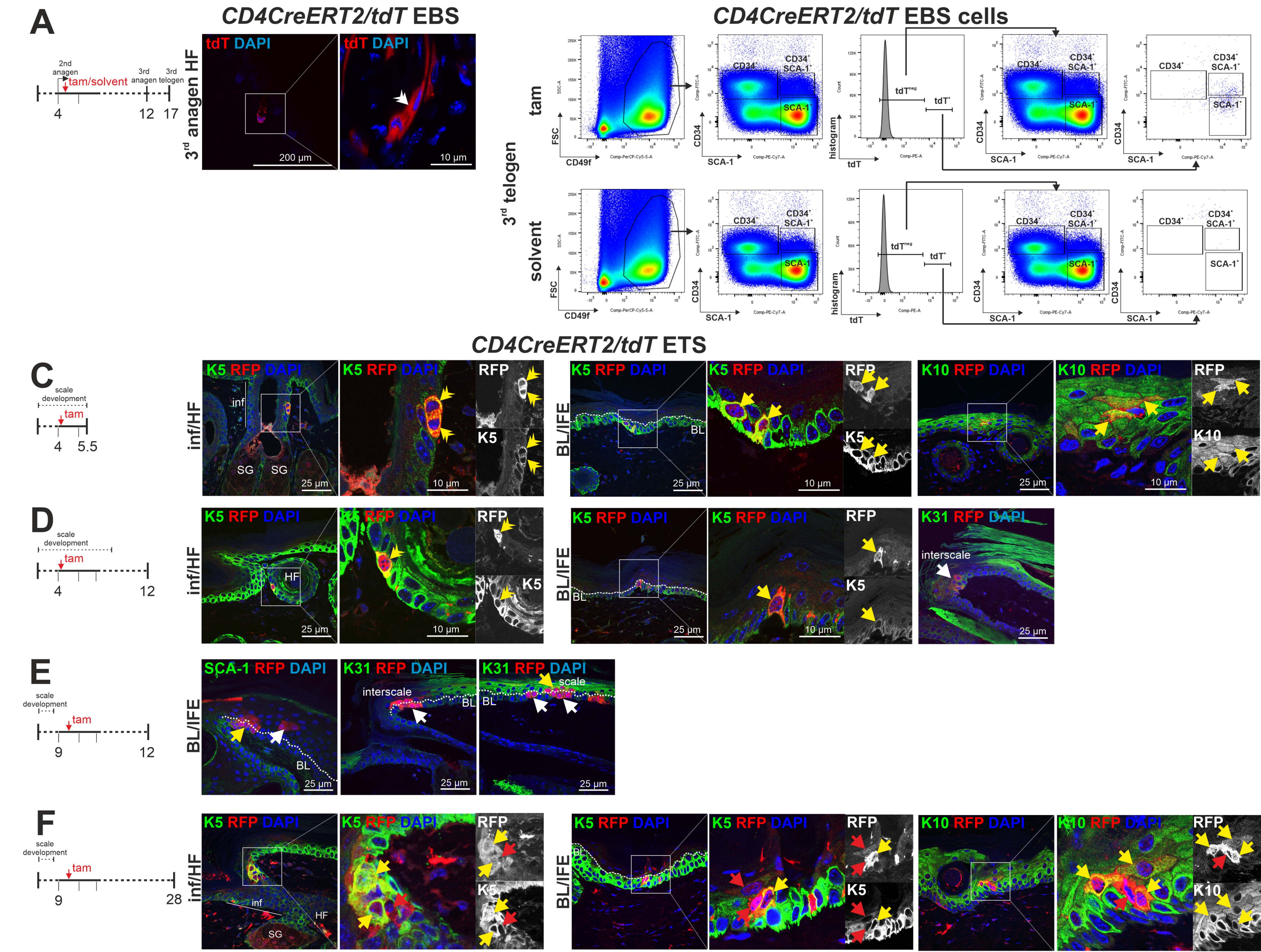

Supplemental Figure S9: Tracking the progeny of CD4$^+$ cells in the EBS and ETS of tamoxifen-induced *CD4CreERT2/tdT* mice.

Analyses of the progeny of CD4$^+$ keratinocytes in the EBS and ETS of *CD4CreERT2/tdT* lineage tracing mice. In the EBS, tamoxifen (tam) application in the 2nd endogenous anagen phase leads to long term tdT labelling of anagen hair follicle and CD49f$^+$ SCA-1$^+$ IFE cells in the telogen skin **(A)**. In the ETS, tam application during scale development results in labelling of K5$^+$ INF and K5$^+$ BL-IFE cells and in labelling of K10$^+$ IFE or K31$^+$ scale IFE cells after 1.5 and 8 weeks **(C,D)**. 8 or 18.5 weeks after tam application after finished scale development leads to tdT-labeling of SCA-1$^+$ BL-IFE and the K31$^+$ scale cells **(E)** or of K5$^+$ INF, K5$^+$ BL-IFE and K10$^+$ interscale IFE cells **(F)**, respectively.

(A) Fluorescent and CD49f/FSC-plotted flow cytometric analyses of a EBS whole mount or EBS cells of tam- or solvent treated *CD4CreERT2/tdT* mice at the 3rd endogenous anagen or the 3rd endogenous telogen phase, respectively. Right: All CD49f$^+$ or tdT^{neg} CD49f$^+$ or tdT$^+$ CD49f$^+$ keratinocytes were SCA-1/CD34-plotted for determination of CD34$^+$ bulge, SCA-1$^+$ IFE and CD34$^+$SCA-1$^+$ epidermal cells (see [11,12]). tdT$^+$ cells were detected in the PE-channel.

(C-F) Immunofluorescent analyses of the ETS of tam-induced *CD4CreERT2/tdT* mice (respective tam regimes are depicted on the left). tdT$^+$ epidermal cells were detected in paraffin sections with antibodies against RFP and K5, K31 or K10.

Nuclei were visualized with DAPI. White double arrowheads: tdT$^+$ hair follicle cells; yellow double arrowheads: double positive tdT$^+$ hair follicle cells; white arrows: tdT$^+$ IFE cells, yellow arrows: double positive tdT$^+$ IFE cells. White dotted lines in (C-F) define the basal layer (BL). White boxes: zoom-in area. HF: hair follicle, inf: infundibulum, SG: sebaceous gland.

SUPPLEMENTAL FIGURE S10

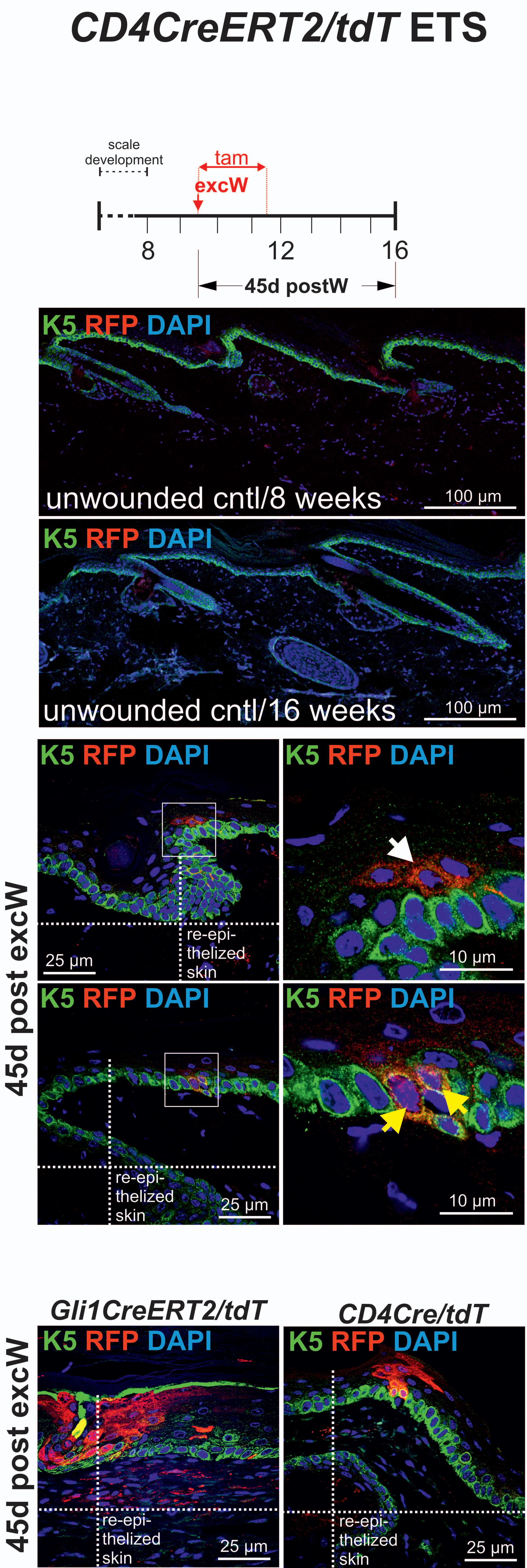

Supplemental Figure S10: Wound-induced colonization of the hair follicle and/or the IFE with the progeny of CD4$^+$ epidermal cells.

Experimental setup and (immune-) fluorescent analyses of unwounded and excW of ETS of tam-injected *CD4CreERT2/tdT* or *Gli1CreERT2/tdT* and *CD4Cre/tdT* control mice. tdT$^+$ IFE cells in wound-near areas in ETS paraffin sections stained with anti-RPF/anti-K5 antibodies 45 days post excW or of unwounded skin. Not any or only very few tdT$^+$ cells were observed in the ETS of untreated, unwounded 8 weeks-old or unwounded 16 weeks-old *CD4CreERT2/tdT* mice, respectively.

Nuclei were visualized with DAPI. tdT expression in paraffin sections was detected using an anti-RFP antibody. White double arrowheads: tdT$^+$ hair follicle cells; yellow double arrowheads: double positive tdT$^+$ hair follicle cells; white arrows: tdT$^+$ IFE cells; yellow arrows: double positive tdT$^+$ IFE cells; WI: wound-induced; postW: post wounding. White boxes: zoom-in areas.

SUPPLEMENTAL FIGURE S11

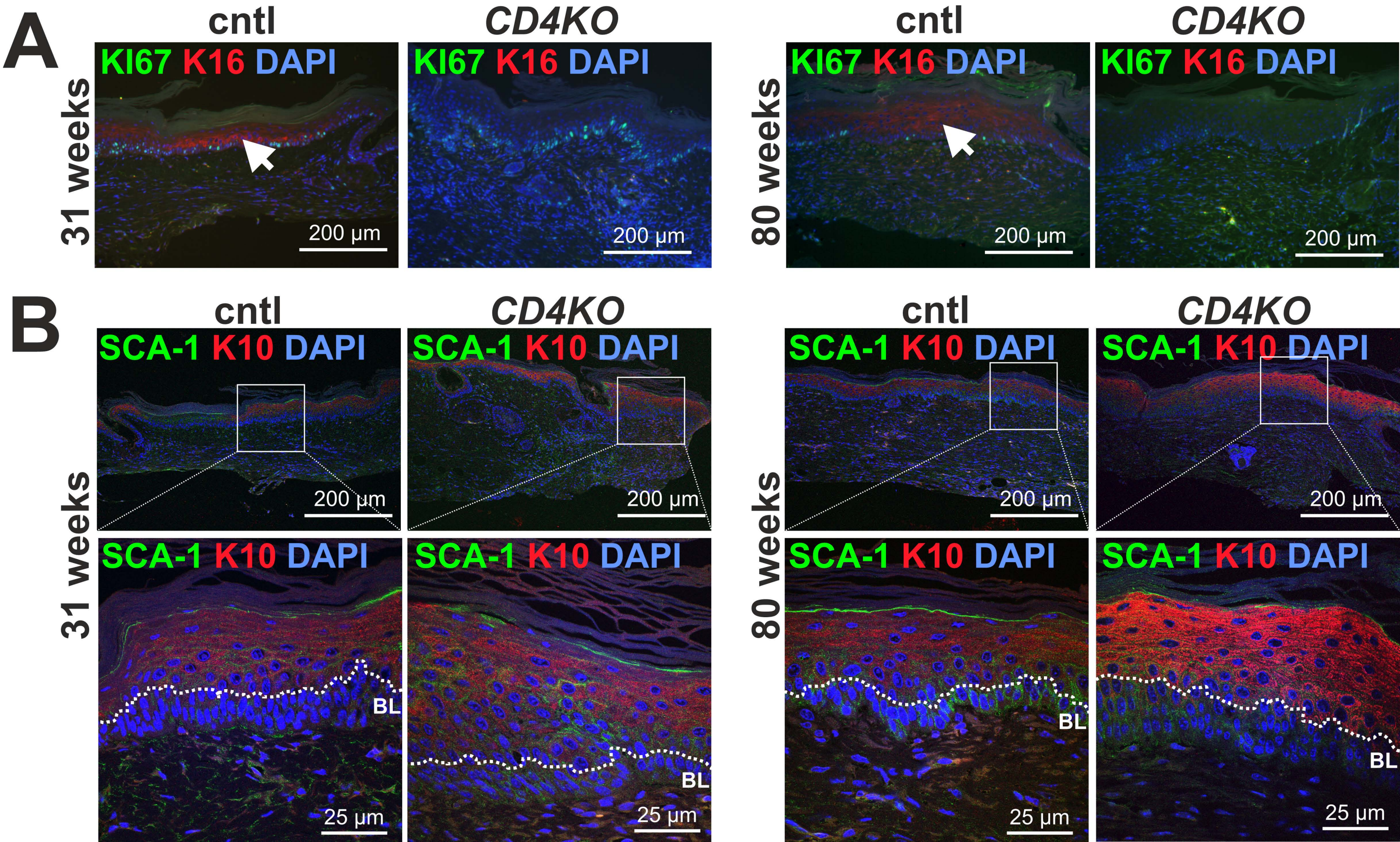

Supplemental Figure S11: Immunohistological analyses of the EBS after excisional wounding of 31 and/or 80 weeks-old *CD4KO* and corresponding cntl mice.

(A,B) Anti-KI67/anti-K16 (A) and anti-SCA-1/anti-K10 (B) antibody-stained paraffin sections of wound-near areas 56d post excW of 31 and 80 weeks old *CD4KO* and age-matched cntl. Nuclei were visualized with DAPI. White arrows: $K16^+$ IFE. White lines in delimit the basal layer (BL). White boxes: zoom-in areas.

SUPPLEMENTAL FIGURE S12

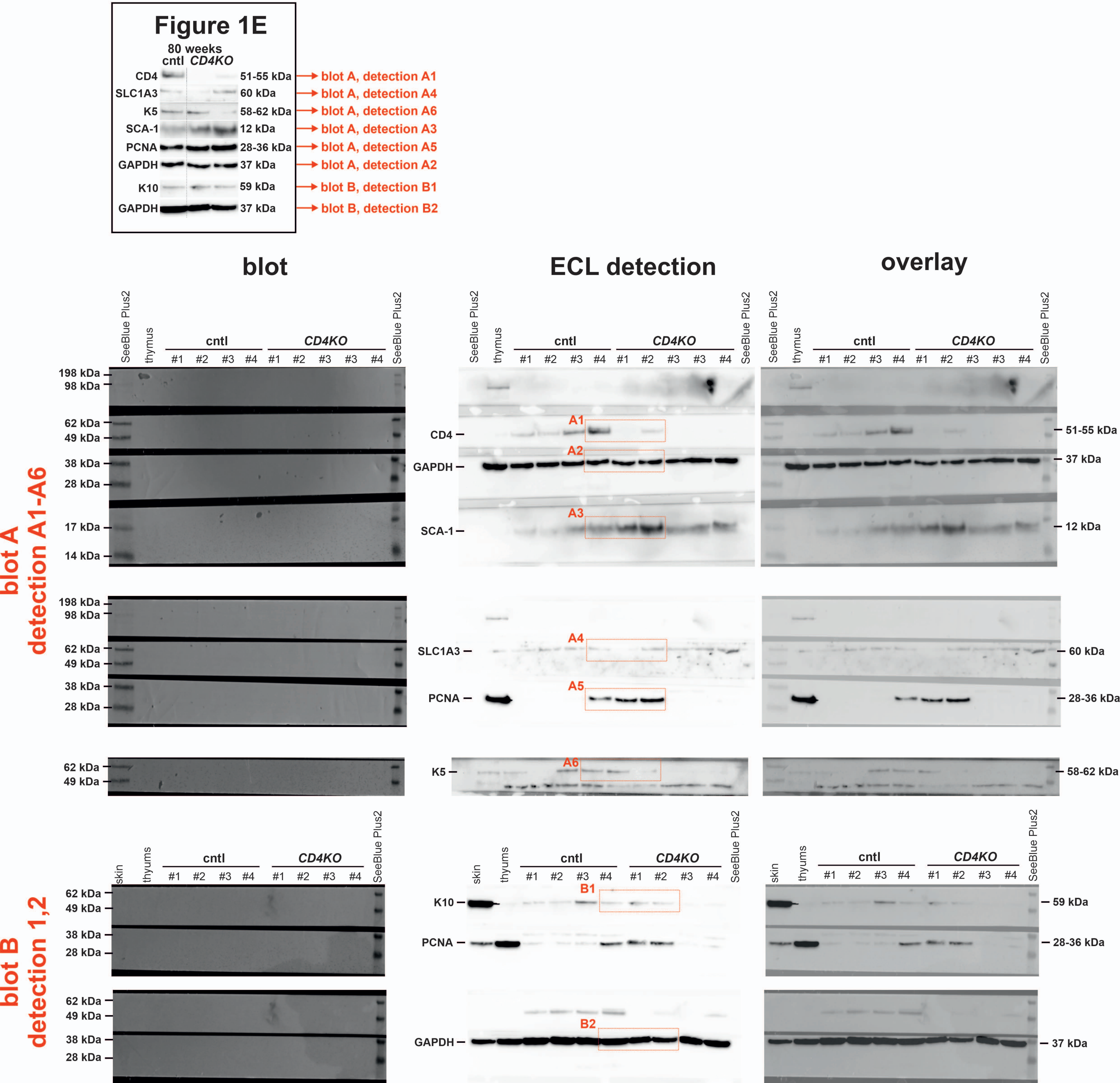

Supplemental Figure S12: Original blots used for Western blot analysis presented in Figure 1E.

The red arrows in the upper section point from the cropped blots shown in Figure 1E to the corresponding original blots and ECL detection. The lower section displays the original blots, ECL detections and overlays. The red boxes indicate the areas of cropping (A1-A6, B1, B2) that are presented in Figure 1E. For Western blotting see Material and method section. SeeBlue Plus2 Prestained Standard for determination of protein sizes was included on each gel/blot. Protein extract from C67BL6/N thymus was simultaneously analyzed as a positive control for CD4 expression and as a negative control for keratinocytes marker expression. Used antibodies and antibody concentrations are summarized in Supplemental Table S10-12.

SUPPLEMENTAL FIGURE S13

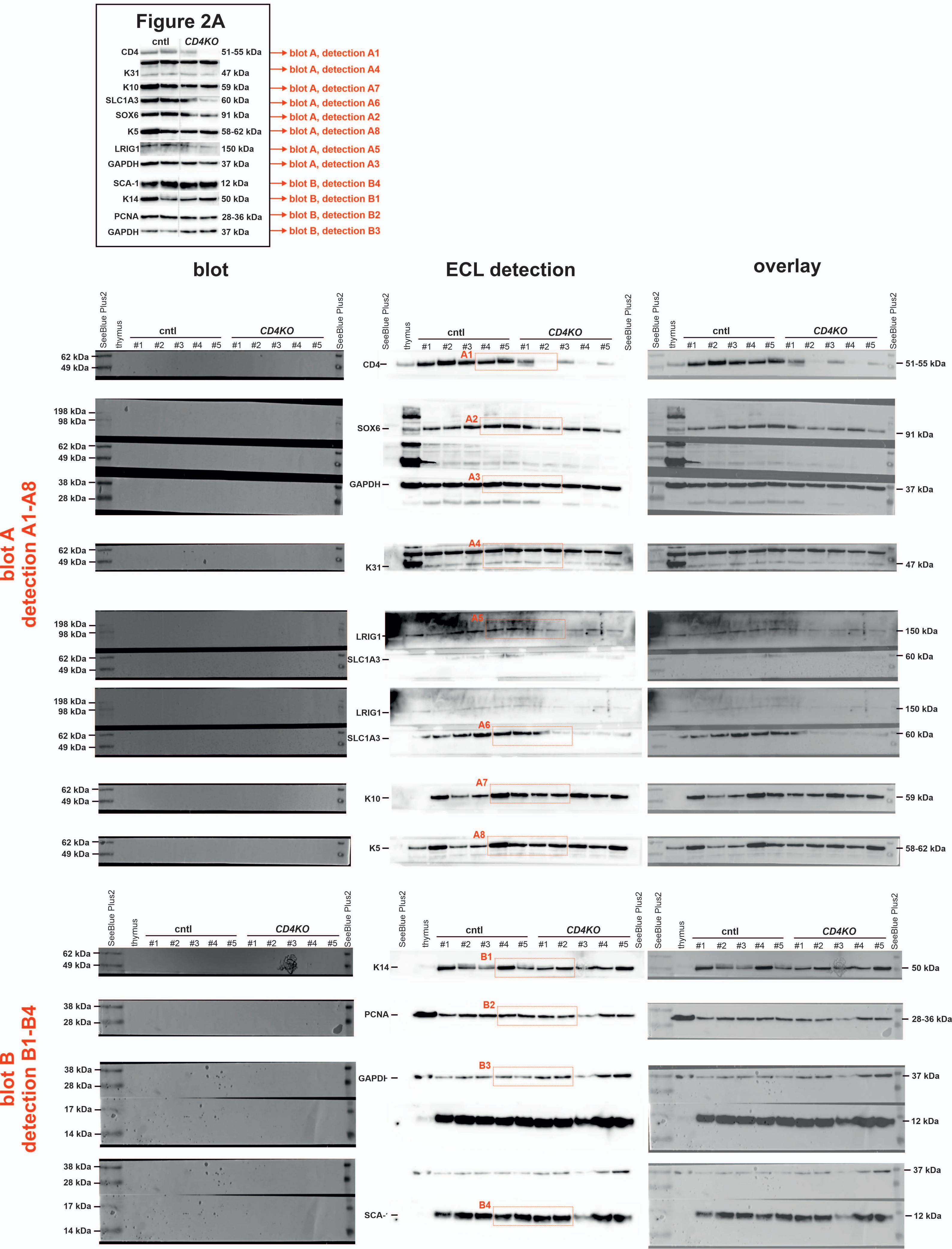

Supplemental Figure S13: Original blots used for Western blot analysis presented in Figure 2A.

The red arrows in the upper section point from the cropped blots shown in Figure 2A to the corresponding original blots and ECL detection. The lower section displays the original blots, ECL detections and overlays. The red boxes indicate the areas of cropping (A1-A8, B1-B4) that are presented in Figure 2A. For Western blotting see Material and method section. SeeBlue Plus2 Prestained Standard for determination of protein sizes was included on each gel/blot. Protein extract from C67BL6/N thymus was simultaneously analyzed as a positive control for CD4 expression and as a negative control for keratinocytes marker expression. Used antibodies and antibody concentrations are summarized in Supplemental Table S10-12.

SUPPLEMENTAL FIGURE S14

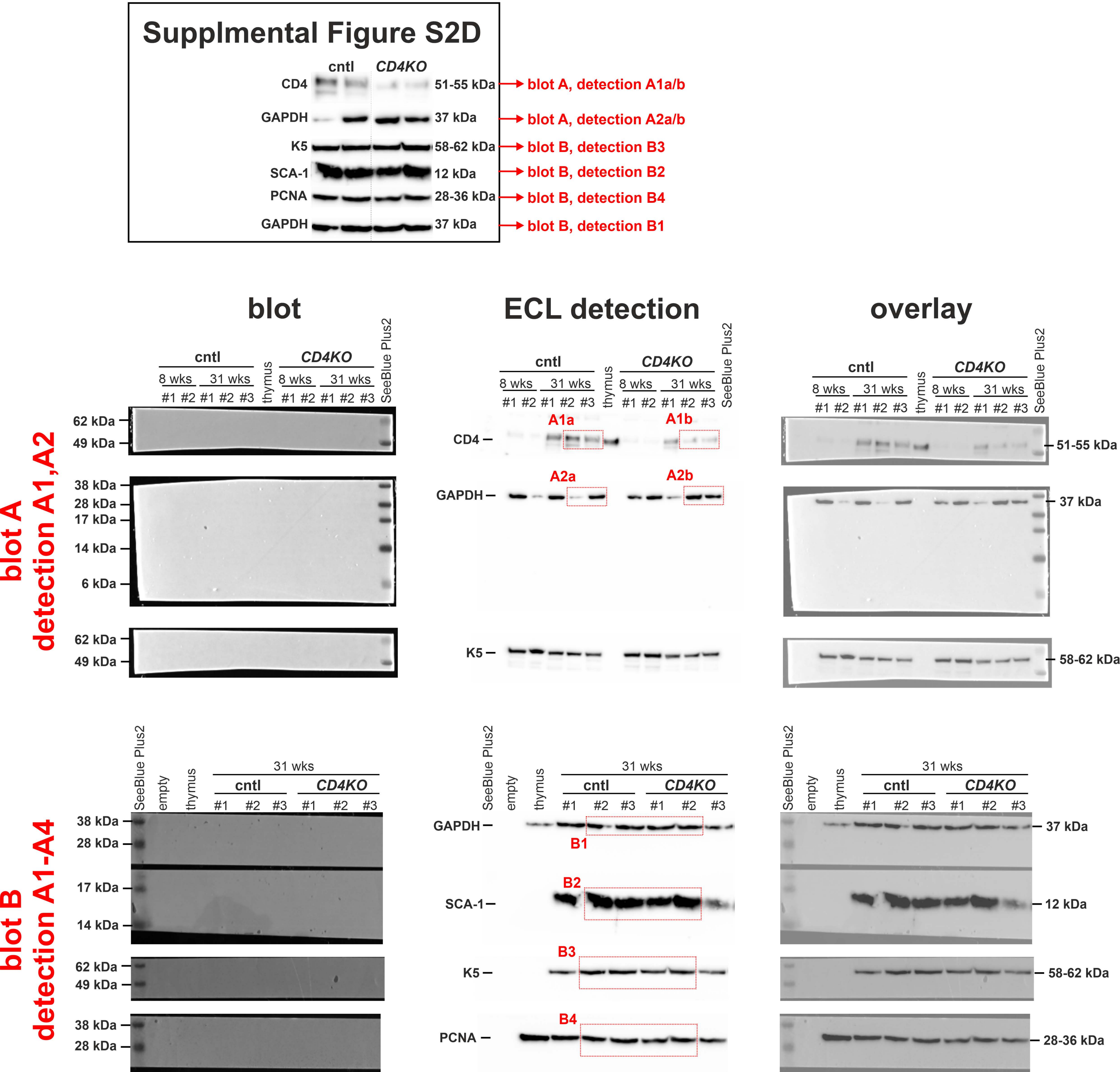

Supplemental Figure S14: Original blots used for Western blot analysis presented in Supplemental Figure S2D.

The red arrows in the upper section point from the cropped blots shown in Supplemental Figure S2D to the corresponding original blots and ECL detection. The lower section displays the original blots, ECL detections and overlays. The red boxes indicate the areas of cropping (A1, A2, B1-B4) that are presented in Supplemental Figure S2D. For Western blotting see Material and method section. SeeBlue Plus2 Prestained Standard for determination of protein sizes was included on each gel/blot. Protein extract from C67BL6/N thymus was simultaneously analyzed as a positive control for CD4 expression and as a negative control for keratinocytes marker expression. Used antibodies and antibody concentrations are summarized in Supplemental Table S10-12.

SUPPLEMENTAL FIGURE S15

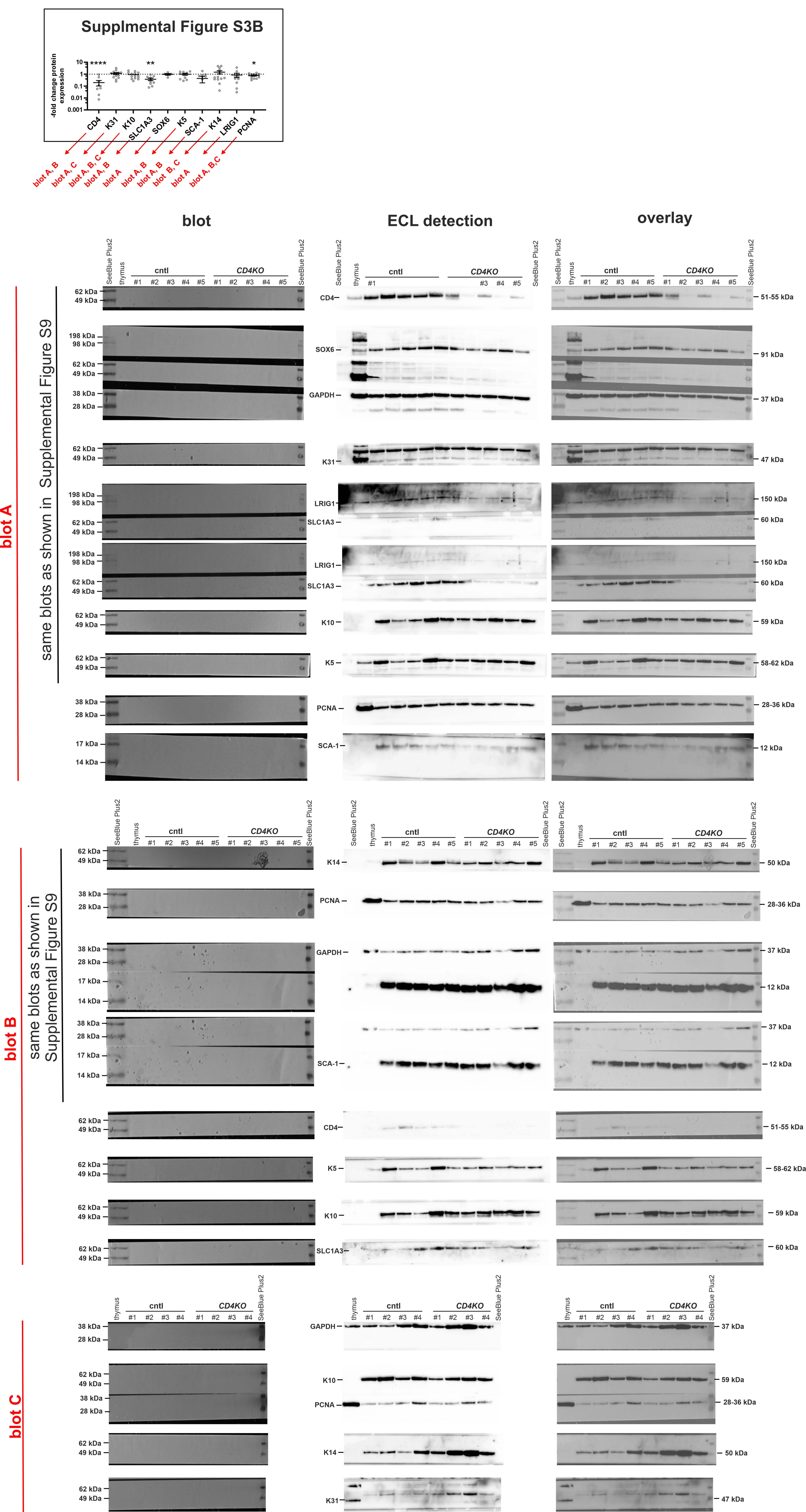

Supplemental Figure S15: Original blots used for quantitative analysis of protein expression presented in Supplemental Figure S3B.

The red arrows in the upper section point from respective protein expression shown in Supplemental Figure S3B to the corresponding original blots and ECL detection. The lower section displays the original blots, ECL detections and overlays. For Western blotting see Material and method section. SeeBlue Plus2 Prestained Standard for determination of protein sizes was included on each gel/blot. Protein extract from C67BL6/N thymus was simultaneously analyzed as a positive control for CD4 expression and as a negative control for keratinocytes marker expression. Used antibodies and antibody concentrations are summarized in Supplemental Table S10-12. Please note that labelled parts of blot A and blot B are identical to cropped blots shown in Figure 2A and to original blots in Supplemental Figure S14.

Supplemental References

1 Uhmann, A. *et al.* DMBA/TPA treatment is necessary for BCC formation from patched deficient epidermal cells in Ptch(flox/flox)CD4Cre(+/-) mice. *The Journal of investigative dermatology* **134**, 2620-2629 (2014). https://doi.org:10.1038/jid.2014.157

2 Brandes, N. *et al.* Spreading of Isolated Ptch Mutant Basal Cell Carcinoma Precursors Is Physiologically Suppressed and Counteracts Tumor Formation in Mice. *International journal of molecular sciences* **21** (2020). https://doi.org:10.3390/ijms21239295

3 Joost, S. *et al.* Single-Cell Transcriptomics Reveals that Differentiation and Spatial Signatures Shape Epidermal and Hair Follicle Heterogeneity. *Cell systems* **3**, 221-237 e229 (2016). https://doi.org:10.1016/j.cels.2016.08.010
